# Ecophysiology and behavioral indicators of thermal stress in freshwater crab *Himalayapotamon emphyseteum* under experimental warming

**DOI:** 10.1242/bio.062895

**Published:** 2026-10-21

**Authors:** Tenzeela, Muhammad Adnan, Aisha Gul, Sanaullah Khan

**Affiliations:** ^1^ Institute of Zoological Sciences, University of Peshawar, Khyber Pakhtunkhwa, Peshawar 2500, Pakistan; ^2^ Department of Zoology, Women University Swabi, Khyber Pakhtunkhwa, Pakistan

**Keywords:** Climate change, Freshwater crab, Thermal tolerance, Metabolic depression, Behavioral thermoregulation, CTMax

## Abstract

Global warming poses substantial challenges to ectothermic organisms, which depend on physiological and behavioral adjustments to maintain homeostasis under thermal stress. We examined the effects of experimental warming on oxygen consumption, ammonia excretion, feeding activity, surface activity, burrow retreat, and critical thermal maximum (CTMax) in *Himalayapotamon emphyseteum*. Crabs were exposed to three independent experimental temperatures (27, 31, and 33°C), and physiological and behavioral responses were assessed. Oxygen consumption rate increased significantly with temperature, from 1.917±0.192 mg O_2_ g^−1^ h^−1^ at 27°C to 3.606±0.631 mg O_2_ g^−1^ h^−1^ at 31°C and 5.835±0.792 mg O_2_ g^−1^ h^−1^ at 33°C (*P*<0.001). Ammonia excretion did not differ between 27 and 31°C but increased significantly at 33°C (*P*<0.001). Feeding rate and surface activity decreased significantly with increasing temperature, whereas burrow retreat increased significantly (*P*<0.001). CTMax was strongly affected by heating rate, increasing from 32.90±0.88°C at 1°C min^−1^ to 34.10±0.74°C at 1°C per 30 min and 35.90±0.32°C at 1°C h^−1^ (*P*<0.001). Experimental warming elicited pronounced physiological and behavioral responses, while measured upper thermal limits were strongly influenced by the heating rate and experimental protocol. Reduced feeding and surface activity, increased burrow retreat, and elevated metabolic rate indicate significant physiological and behavioral responses to warming.

## Teaser

**Summary:** Rising temperatures substantially alter metabolism and behavior in *Himalayapotamon emphyseteum*, revealing physiological sensitivity to warming and strong methodological effects of heating rate on CTMax.

## INTRODUCTION

Rising average temperatures and extreme temperatures are occurring as a result of climate change, with significant implications for the body temperature and physiology of ectothermic organisms. As for the regulation of body temperature, ectotherms rely heavily on environmental temperatures to control metabolic rate, activity level, and energy budget; they are often considered to be one of the most susceptible groups to current warming trends and heightened temperature variability (Pörtner, 2010; Sokolova, 2021). Aquatic ecosystems may be particularly vulnerable, as increasing temperature can simultaneously decrease water oxygen levels, increase energy requirements, and modify habitat suitability, thereby impacting survival, distribution and ecosystem functioning of aquatic organisms (Woodward et al., 2010; Sokolova, 2021).

In aquatic ectotherms, temperature affects a range of physiological processes, including the consumption of oxygen, energy budget, the rates of biochemical reactions, and nitrogen recycling (Clarke and Fraser, 2004; Carter et al., 2023). In crustaceans, metabolic rates generally increase with rising temperature up to a species-specific peak and then decline as thermal tolerance limits are reached and aerobic performance becomes limited (Pörtner, 2010; Ern et al., 2020). Oxygen uptake is typically used as a measure of metabolic rate and aerobic capacity, and ammonia release can be used to supplement data on oxygen consumption with insight into protein metabolism, the production of nitrogen waste and metabolic imbalance during stress (Widdows and Johnson, 1988; Sokolova, 2021). Together, these measurements can present valuable data on the impact of thermal stress on physiological integrity and energy allocation.

Warming responses are commonly quantified by the Q_10_ coefficient, which represents the change in the rate of a physiological process for a 10°C increase in temperature. While Q_10_ is commonly used in thermal physiology, it can vary considerably across species, acclimation states and environments, highlighting the need for species-specific assessment of warming responses (Clarke and Fraser, 2004; Carter et al., 2023). Acute tolerance to high temperatures is often measured using the critical thermal maximum (CTMax), which is the temperature at which an animal's coordinated motor function fails due to heat-induced physiological dysfunction (Lutterschmidt and Hutchison, 1997; Ern et al., 2020). Although CTMax provides a good measure of acute thermal tolerance, it is not sufficient in predicting ecological outcomes, since critical sublethal changes in metabolic, behavioral and performance responses may occur well before lethal thresholds (Sunday et al., 2014; Sokolova, 2021).

Changes in behavior are one of the earliest indicators of thermal stress and may play a critical role in ecology. Among freshwater crabs, changes in feeding, walking, and avoiding behavior (e.g. burrow retreat) may act as immediate mechanisms to prevent thermal stress and achieve physiological homeostasis. However, such responses can also have ecological and energetic consequences by restricting foraging opportunities and food intake and altering activity patterns, potentially affecting growth, reproduction and long-term fitness (Crowther and Schwanz, 2025; Sunday et al., 2014). While freshwater crabs are known to play vital roles as benthic consumers, detritivores, and bioturbators in streams, little research has been conducted on the collective physiological and behavioral responses of freshwater crabs to projected temperature rises, especially in South Asian freshwater ecosystems.

In this context, the current study explores ecophysiological responses of the freshwater crab *Himalayapotamon* sp., from a rocky stream in Pakistan, under ambient and experimentally induced temperature conditions. In particular, it measures rates of oxygen consumption, ammonia excretion, temperature sensitivity (Q_10_), CTMax, and several behavioral traits, such as feeding, surface locomotion, and retreat into burrows. Based on the preceding literature, we tested the following predictions: (1) oxygen consumption rate (OCR) would increase with temperature up to a species-specific thermal optimum, after which it may plateau or decline; (2) ammonia excretion would increase at higher temperatures, reflecting elevated protein catabolism; (3) warming would suppress feeding and surface activity while increasing burrow retreat as behavioral thermoregulation; (4) CTMax would be influenced by heating rate, with slower heating rates potentially allowing thermal acclimation and thus higher tolerance or, alternatively, allowing cumulative heat damage and thus lower tolerance; and (5) moderate warming (4°C above ambient) would induce significant sublethal effects on both physiology and behavior, indicating that crabs may experience ecological impacts well below their lethal thermal limits.

This work combines physiological and behavioral measures to give a more holistic picture of thermal tolerance and sensitivity in this species and to yield insight into how freshwater crabs may respond to current and future temperature rises.

## RESULTS

### Physiological response

Temperature strongly affected OCR in *Himalayapotamon emphyseteum* (Fig. 1A, Table 1). Because the variances differed among temperature treatments (Levene's test, *P*<0.001), differences were evaluated using Welch's one-way analysis of variance (ANOVA). OCR differed significantly among temperatures (Welch's F_2,16.63_=162.31, *P*<0.001). Mean OCR increased from 1.917±0.192 mg O_2_ g^−1^ h^−1^ at 27°C to 3.606±0.631 mg O_2_ g^−1^ h^−1^ at 31°C and 5.835±0.792 mg O_2_ g^−1^ h^−1^ at 33°C (mean±s.d., *n*=12 independent crabs per treatment). Games–Howell post hoc comparisons showed significant differences between all three temperatures (all *P*<0.001). Thus, OCR increased progressively across the entire experimental warming range. Relative to 27°C, mean OCR was approximately 88% higher at 31°C and 204% higher at 33°C.

**Fig. 1.**
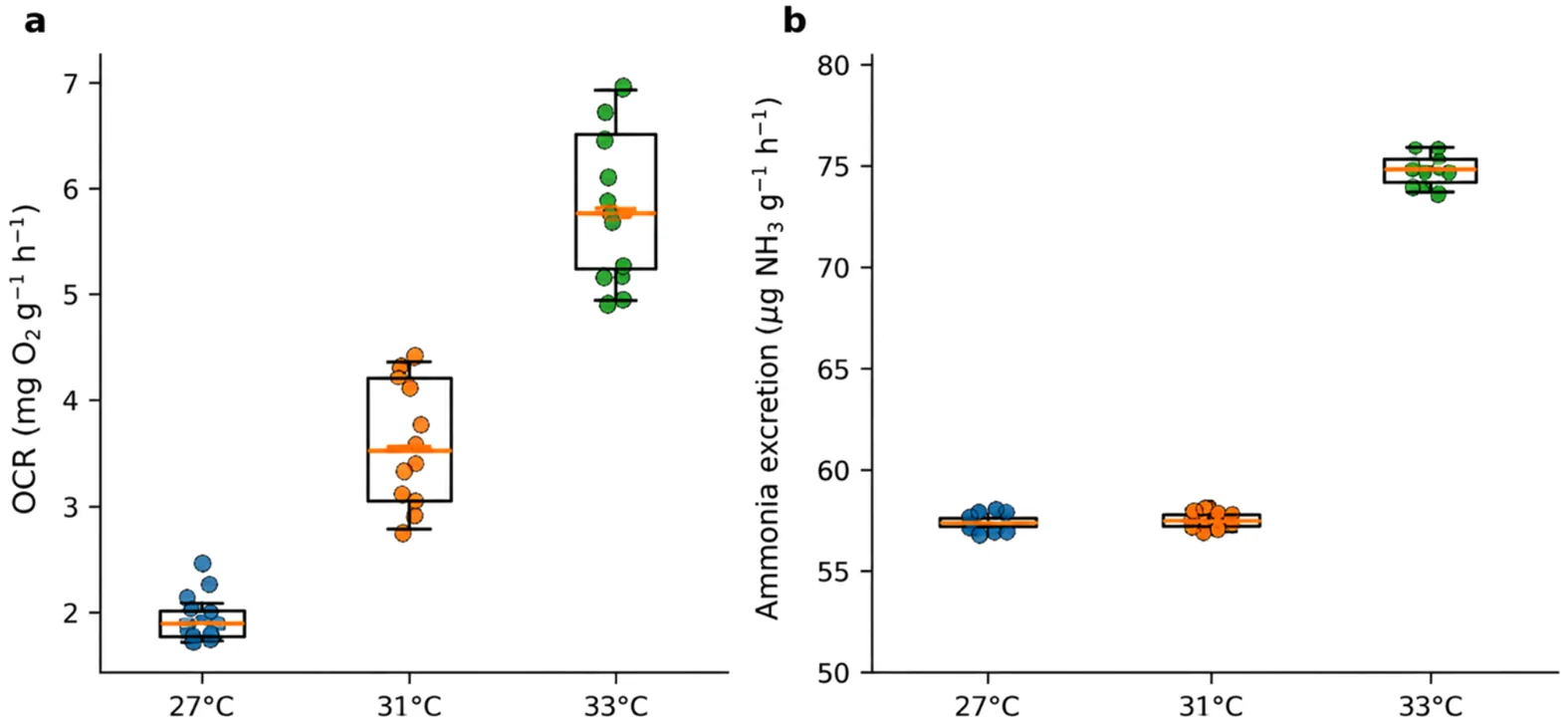
**Physiological responses of *H. emphyseteum* to temperature.** (A) OCR and (B) ammonia excretion rate at 27, 31 and 33°C. Individual observations are shown as points, boxes indicate the interquartile range, horizontal lines indicate medians, and thick horizontal marks indicate group means. *n*=12 per temperature.

**Table 1.** Values are mean±s.d.; *n*=12 independent crabs per temperature treatment

Parameter	27°C (*n*=12)	31°C (*n*=12)	33°C (*n*=12)
Oxygen consumption rate (mg O_2_ g^−1^ h^−1^)	1.917±0.192^a^	3.606±0.631^b^	5.835±0.792^c^
Ammonia excretion rate (μg NH_3_ g^−1^ h^−1^)	57.568±0.013^a^	57.582±0.049^a^	75.088±0.042^b^
Feeding rate (claw-to-mouth feeding events per minute)	14.33±1.15^a^	8.33±0.65^b^	2.67±0.49^c^
Surface activity (min h^−1^)	51.67±1.15^a^	40.50±1.00^b^	11.58±1.83^c^
Burrow retreat (min h^−1^)	6.83±0.72^a^	14.33±1.15^b^	36.67±3.37^c^

Different superscript letters within a row indicate significant differences among temperature treatments. Tukey's HSD was used for ammonia excretion, feeding rate and surface activity; Games–Howell comparisons were used for OCR and burrow retreat because of unequal variances.

Similarly, ammonia excretion also differed significantly among temperature treatments (Fig. 1B, Table 1; one-way ANOVA, F_2,33_=862,100.19, *P*<0.001). Ammonia excretion was 57.568±0.000013 μg NH_3_ g^−1^ h^−1^ at 27°C, 57.582±0.000049 μg NH_3_ g^−1^ h^−1^ at 31°C, and 75.088±0.000042 μg NH_3_ g^−1^ h^−1^ at 33°C (*n*=12 per treatment). Tukey's honestly significant difference (HSD) indicated no significant difference between 27 and 31°C (*P*=0.665), whereas ammonia excretion at 33°C was significantly higher than at both 27 and 31°C (both *P*<0.001).

This trend indicates that unlike OCR, which increased progressively with temperature, ammonia excretion remained essentially unchanged between 27 and 31°C and increased substantially only at 33°C.

### Behavioral responses

Temperature significantly affected feeding rate (Fig. 2A, Table 1; one-way ANOVA, F_2,33_=612.67, *P*<0.001). Feeding rate decreased from 14.33±1.15 claw-to-mouth feeding events per minute at 27°C to 8.33±0.65 claw-to-mouth feeding events per minute at 31°C and 2.67±0.49 feeding events per minute at 33°C (*n*=12 independent crabs per treatment). Tukey's HSD indicated significant differences among all three temperatures (all *P*<0.001). Compared with 27°C, the feeding rate was approximately 42% lower at 31°C and 81% lower at 33°C.

**Fig. 2.**
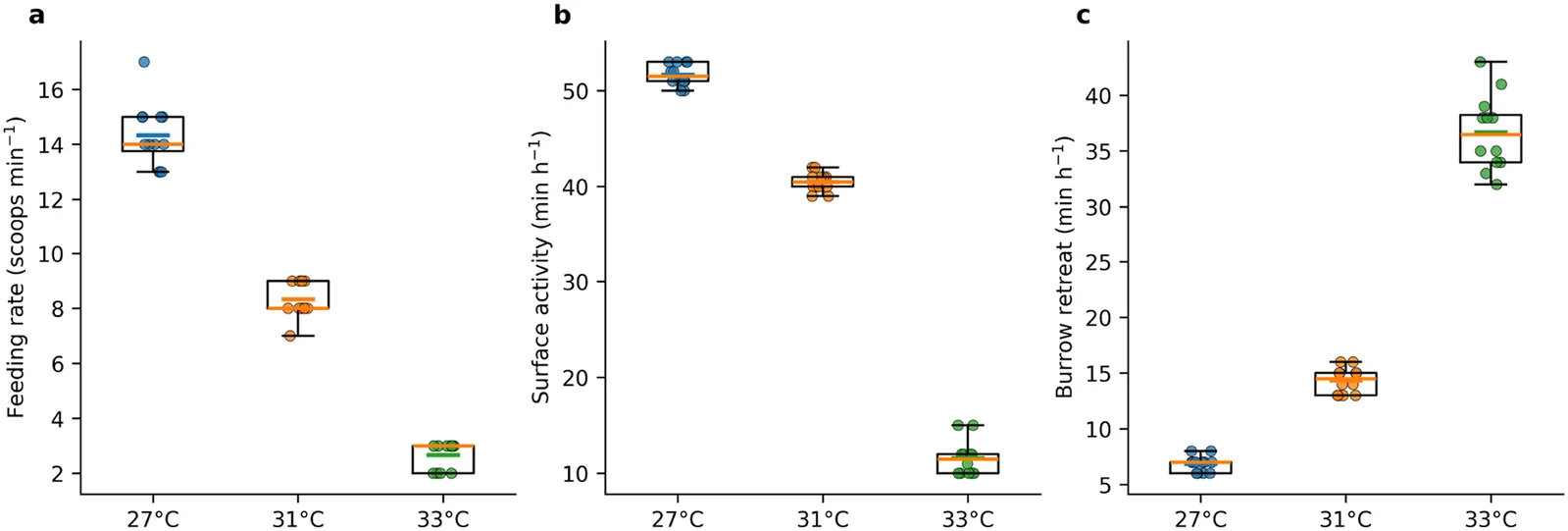
**Behavioral responses of *H. emphyseteum* to temperature.** (A) Feeding rate, (B) surface activity and (C) burrow retreat at 27, 31 and 33°C. Individual observations are shown as points, boxes indicate the interquartile range, horizontal lines indicate medians, and thick horizontal marks indicate group means. *n*=12 per temperature. All pairwise temperature comparisons were significant (Tukey's HSD, *P*<0.001).

Surface activity also decreased significantly with increasing temperature (Fig. 2B, Table 1; one-way ANOVA, F_2,33_=2707.71, *P*<0.001). Mean surface activity decreased from 51.67±1.15 min h^−1^ at 27°C to 40.50±1.00 min h^−1^ at 31°C and 11.58±1.83 min h^−1^ at 33°C (*n*=12 per treatment). Tukey's HSD showed significant differences among all three temperature treatments (all *P*<0.001). Surface activity was approximately 78% lower at 33°C than at 27°C.

In contrast, burrow retreat increased strongly with temperature (Fig. 2C, Table 1). Because the variances differed significantly among treatments (Levene's test, *P*<0.001), Welch's ANOVA was used. Burrow retreat differed significantly among temperatures (Welch's F_2,18.78_=557.34, *P*<0.001). Mean retreat time increased from 6.83±0.72 min h^−1^ at 27°C to 14.33±1.15 min h^−1^ at 31°C and 36.67±3.37 min h^−1^ at 33°C (*n*=12 per treatment). Games–Howell comparisons indicated significant differences between all three temperatures (all *P*<0.001). Overall, warming was associated with a coordinated reduction in feeding and surface activity and a marked increase in time spent retreating into the burrow.

### Correlation between OCR and behavioral measurement

Pearson's correlation analyses were performed independently for each experimental temperature to investigate the relationship between physiological (OCR) and behavioral responses. For surface activity, OCR was not significantly correlated at 27°C (*r*=0.274, *P*=0.389), 31°C (*r*=−0.088, *P*=0.785), or 33°C (*r*=−0.153, *P*=0.635). For burrow retreat, OCR showed a significant negative correlation at 27°C (*r*=−0.594, *P*=0.042); however, this relationship was not observed at 31°C (*r*=−0.193, *P*=0.547) or 33°C (*r*=0.021, *P*=0.947). Overall, these results do not support a consistent relationship between OCR and behavioral responses across the tested temperature range (Fig. 3).

**Fig. 3.**
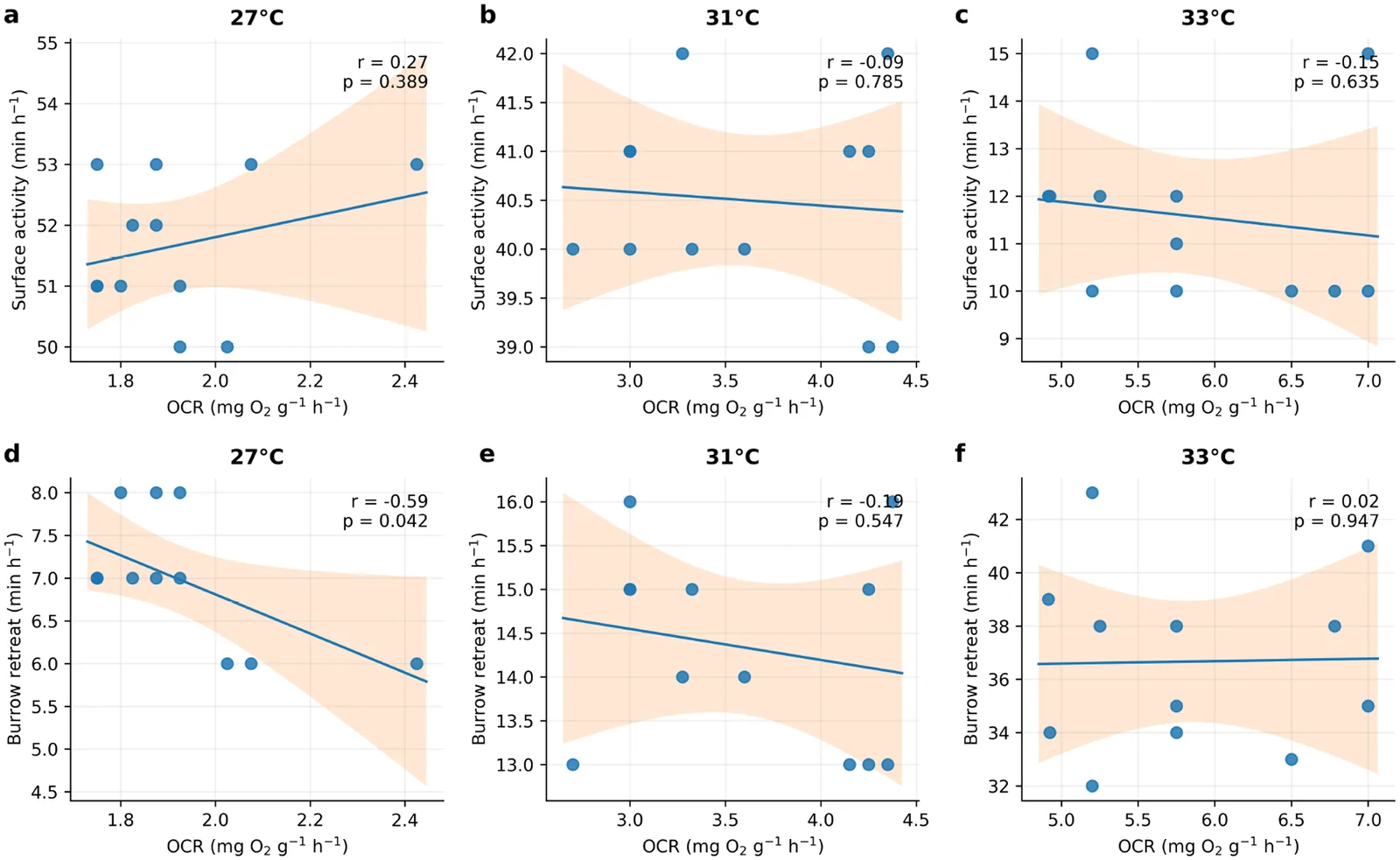
**Relationships between OCR and behavioral responses at 27, 31, and 33°C.** (A–C) Relationships between OCR and surface activity and (D–F) relationships between OCR and burrow retreat. Points represent individual crabs, solid lines represent linear regression fits, and shaded bands indicate 95% CIs. Pearson's correlation coefficients (*r*) and associated *P*-values are shown in each panel.

### Thermal coefficient (Q_10_)

Oxygen consumption exhibited pronounced temperature sensitivity across the experimental range. The estimated Q_10_ was 4.86 [95% confidence interval (CI): 3.67–6.29] between 27 and 31°C and 6.40 (95% CI: 5.48–7.40) between 27 and 33°C (Table 2). These results indicate a strong increase in metabolic demand with increasing temperature.

**Table 2.** Q_10_ values were calculated from OCR between 27–31 and 27–33°C

S. No.	Temperature interval	Value	95% CI
1	Q_10_ (27–31°C)	4.86	(3.67–6.29)
2	Q_10_ (27–33°C)	6.40	(5.48–7.40)

The 95% CIs were estimated by bootstrap resampling of the individual OCR observations.

### CTMax

CTMax differed significantly among heating rates (one-way ANOVA: F_2,27_=30.50, *P*<0.001, η^2^=0.693; Fig. 4, Table 3). Mean CTMax was 35.60±0.70°C at 1°C min^−1^, 34.10±0.74°C at 1°C per 30 min, and 32.90±0.88°C at 1°C h^−1^ (*n*=10 per heating rate). Post hoc comparisons (Tukey's HSD) revealed significant differences among all three heating rates (1°C min^−1^ versus 1°C per 30 min, *P*=0.0005; 1°C min^−1^ versus 1°C h^−1^, *P*<0.001; 1°C per 30 min versus 1°C h^−1^, *P*=0.0049). Overall, CTMax decreased progressively as the heating rate decreased, with the highest values recorded at the fastest heating rate.

**Fig. 4.**
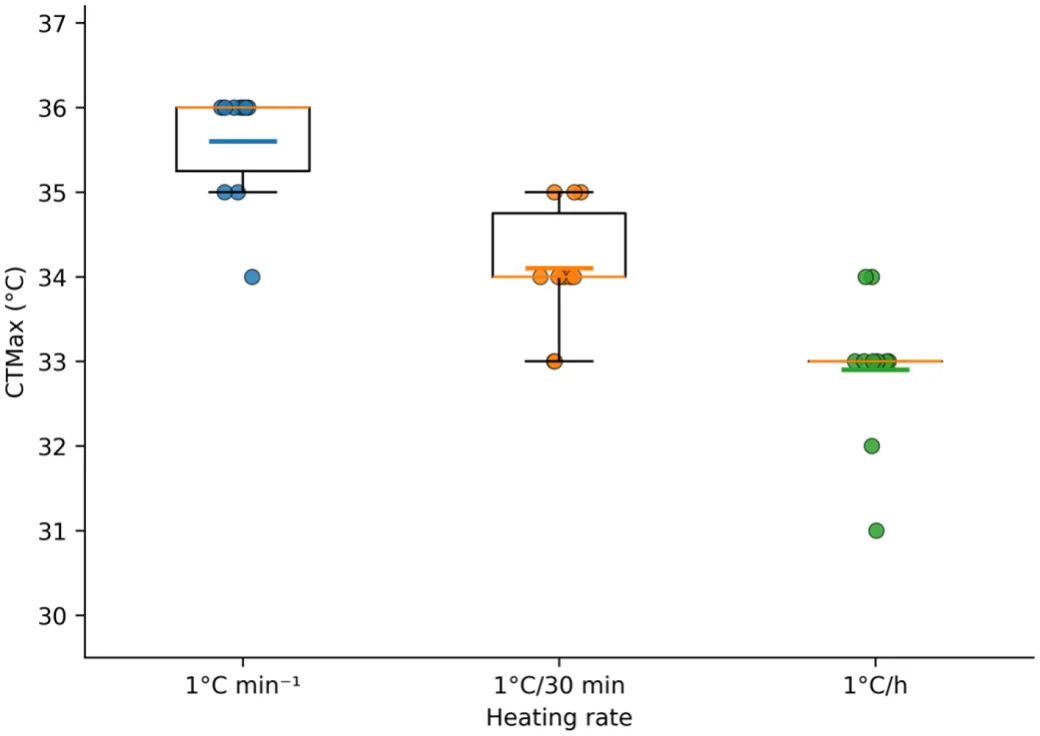
**CTMax at different heating rates.** Individual observations are shown as points, boxes indicate the interquartile range, horizontal lines indicate medians, and horizontal marks indicate group means. Ten independent individuals were tested at each heating rate. Tukey's HSD test (*P*<0.05).

**Table 3.** Values are mean±s.d.

S. No.	Heating rate	CTMax (°C)
1	1°C min^−1^ (*n*=10)	35.60±0.70^a^
2	1°C per 30 min (*n*=10)	34.10±0.74^b^
3	1°C h^−1^ (*n*=10)	32.90±0.88^c^

Different superscript letters indicate significant differences among heating rates according to Tukey's HSD test (*P*<0.05).

One-way ANOVA: F_2,27_=30.50, *P*<0.001, η^2^=0.693. Ten independent individuals were tested at each heating rate.

## DISCUSSION

Freshwater ectotherms are viewed as being extremely sensitive to the current global increase in mean temperatures, but unfortunately, detailed experimental evidence of both physiological and behavioral thresholds of ectothermic species is astonishingly scant, particularly with respect to understudied regions such as South Asia (Sayer et al., 2025). The current work provides a multi-trait analysis of how stream-dwelling *H. emphyseteum* responds to climate warming scenarios expected by the end of the century. We find that even below the lethal thresholds, the crab is subjected to strong metabolic disturbances, a change in the excretion of nitrogen and a sharp reorganization of its activity and refuge-seeking behavior. All these changes are indicative of a thermally induced trade-off between short-term survival and long-term energy balance and suggest that behavioral thermoregulation may have a high fitness cost in future climate conditions (Hews et al., 2021).

OCR increased progressively across the experimental temperature range, reaching its highest value at 33°C (5.835±0.792 mg O_2_ g^−1^ h^−1^). This pattern indicates that warming substantially increased metabolic demand in *H. emphyseteum*. The positive relationship between temperature and metabolic rate is consistent with the general temperature dependence of physiological processes in ectotherms, in which increasing temperature accelerates biochemical reactions and increases the energetic demands of maintaining organismal function (Clarke and Fraser, 2004).

The magnitude of the metabolic response was further reflected in the high Q_10_ values calculated for OCR, with values of 4.86 between 27 and 31°C and 6.40 between 27 and 33°C. These values indicate pronounced thermal sensitivity of OCR over the experimental temperature range. However, Q_10_ estimates should be interpreted within the range over which they were calculated and should not be used to extrapolate metabolic responses beyond the temperatures tested. Similarly, the large increase in OCR between 27 and 33°C indicates a substantial biological response to warming but, by itself, does not establish a thermal optimum or proximity to an upper thermal limit (Padilla-Ramírez et al., 2015).

Importantly, OCR increased continuously from 27 to 33°C, and no plateau or decline in metabolic rate was observed. Consequently, the present data do not allow reconstruction of a complete thermal performance curve or identification of an optimal temperature for metabolic performance. A complete assessment of the thermal response would require measurements across a broader temperature range, particularly at temperatures above 33°C, where metabolic rate may eventually plateau or decline. Therefore, interpretations based on metabolic depression, a transition to anaerobic metabolism, or oxygen limitation cannot be directly inferred from the present OCR data.

The continuous increase in OCR is consistent with the expectation that warming elevates metabolic oxygen demand in ectotherms. However, increased metabolic demand alone does not demonstrate that oxygen supply becomes limiting. The oxygen- and capacity-limited thermal tolerance framework proposes that thermal limits can emerge when oxygen demand exceeds the capacity of oxygen uptake and delivery systems, but the general applicability of this mechanism across ectotherms remains debated and may be species dependent and context dependent (Pörtner et al., 2017; Verberk et al., 2016). Because oxygen availability, aerobic scope, cardiovascular performance, or anaerobic metabolism was not measured in the present study, the role of oxygen limitation in determining thermal tolerance in *H. emphyseteum* cannot be established.

The rate of excretion of ammonia was constant between 27 and 31°C; however, it increased by approximately 30% at 33°C. This pattern suggests that nitrogen balance in the crab up to 31°C is maintained by routine protein turnover, and at 33°C, there is a transition to a more intense protein catabolism, which is likely to meet an increase in metabolic requirements as aerobic pathways are nearly saturated (Sokolova, 2021). The highest ammonia concentrations observed (0.40 mg l^−1^ at 33°C) are well below the reported acute toxicity threshold for freshwater crustaceans [(lethal concentration) LC_50_ values typically >1.0 mg NH_3_ l^−1^; USEPA, 1999] and are unlikely to have influenced OCRs. However, we acknowledge that prolonged exposure to even low ammonia concentrations could potentially interact with thermal stress, and future studies should consider this interaction. The decoupling between OCR and ammonia excretion (OCR increased gradually while ammonia increased only at the highest temperature) indicates that the crab initially relies on lipid and carbohydrate sources; only at extremely high temperatures does it have to shift to the breakdown of proteins. This energetically inefficient response can result in tissue wasting in case of prolonged exposure (da Silva Vianna et al., 2020). These same patterns have been observed in other crustaceans, such as fiddler crabs and blue crabs (García-Rueda et al., 2021). Multiple studies show mixed responses for ammonia excretion under temperature fluctuation, with substantial species-specific variation and condition-dependent responses (Devine and Vanni, 2002; Madeira et al., 2019). For instance, a meta-analysis conducted by Oliveira-Cunha, 2026) reported that in freshwater fish, nitrogen excretion generally increases with increasing temperature. However, this trend is not uniform across species. Small planktonic crustacean *Daphnia magna* at an elevated temperature (5°C increase) showed a decreased ammonia excretion rate (AER) while simultaneously increasing urea excretion, suggesting a shift in nitrogen metabolism and excretion pathway rather than direct ammonia influx (Nash et al., 2022). Similarly, in freshwater mussels, *Elliptio complanata* showed variation in response to temperature fluctuation, while other species such as *Amblema plicata*, *Fusconaia flava* and *Lampsilis cardium* followed an inconsistent pattern of ammonia excretion (Ganser et al., 2015). The evidence confirms species-specific variation in thermal responses (Leiva et al., 2024) but contradicts the claim of consistently elevated ammonia excretion at high temperatures across organisms.

The most sensitive thermal stress indicators were behavioral responses. At 33°C, the rate of feeding was reduced by 85%, surface activity was decreased by 78%, and the burrow retreat increased by six times. These adaptations are typical thermoregulatory strategies: crabs retreat into cooler, wet burrows to avoid deadly surface temperatures (Hews et al., 2021). These findings are supported by several other studies which demonstrate that temperature fluctuation triggers thermoregulatory adaptation in crabs' behavior, reducing foraging and increasing burrow retreat. Vianna et al. (2019) evaluated two fiddler crab species (*Leptuca uruguayensis* and *Leptuca leptodactyla*) at 33°C and reported increased burrow retreat time in both species, with reduced foraging activity in *L uruguayensis.* Similarly, Brodie et al. (2023) recorded a clear decline in mating and foraging in *Minuca pugnax* males under an experimental temperature of 32°C, where the animal spent more time underground, which automatically compromises courtship and active foraging. This idea is further strengthened by Hews et al. (2021) through their experiment on the body temperature of Atlantic marsh fiddler crab *M. pugnax* and burrow retreat. This study revealed burrow retreat in crabs with 24°C body temperature, a point far below lethal body temperature, indicating fitness trade-offs. This result suggests that temperature elevation could impact animals far earlier in the year than expected. However, there is a gap in long-term fitness or survival data that quantify the actual outcomes of these trade-offs at the population level. The behavioral mechanism and immediate fitness trade-offs are strongly supported by different studies (Willett, 2010; Barley et al., 2021; Kirk et al., 2023), yet the long-term energy compromise and survival in food-deficient environments remain unexplored. While burrow retreat provides immediate thermal refuge and protection from predators and desiccation, extended periods spent in burrows may indirectly increase predation risk in at least two ways: (1) by reducing time available for vigilance and escape responses if the crab must eventually emerge to forage and (2) by increasing the risk of being trapped in burrows during flash floods or habitat disturbances, which are common in the rocky streams of northern Pakistan. Additionally, in fast-flowing streams with limited structural complexity, crabs that retreat into burrows may reduce their ability to detect approaching predators compared to individuals maintaining surface activity. Furthermore, extended burrow retreat reduces mate encounters and foraging opportunities, with potential long-term consequences for population fitness.

The most ecologically significant finding of this study is the simultaneous occurrence of increased metabolic expenditure and reduced energy acquisition under warming. At 33°C, OCR was approximately three times that at 27°C (5.835 versus 1.917 mg O_2_ g^−1^ h^−1^), representing a 204% increase in metabolic demand. Concurrently, the feeding rate decreased by 81% (from 14.33 to 2.67 events per minute) and surface activity by 78% (from 51.67 to 11.58 min h^−1^). This creates a potentially severe energetic mismatch: crabs must meet substantially higher maintenance costs while having drastically reduced opportunities to acquire energy.

Experimental warming resulted in obvious changes in physiological and behavioral responses, but there was no consistent relationship between the individual OCR and behavioral traits at any temperature for the correlation analysis. No significant correlations were found for OCR with any other surface activity; however, a significant correlation was found for OCR with burrow retreat only at 27°C (*r*=−0.594, *P*=0.042). This relationship was not sustained at higher temperatures, so it should be interpreted cautiously. The data presented here indicate that both metabolic and behavioral reactions to temperature occur at the treatment level but that there may be inter-individual differences in these variables without a direct correlation. This inter-individual metabolic diversity has been known for a long time and may lead to a complex phenotype–metabolism relationship (Pettersen et al., 2018; Auer et al., 2016).

The results of the CTMax revealed a clear positive relationship between the heating rate and the thermal tolerance: the faster the ramping (1°C min^−1^), the higher the CTMax (35.5°C). This pattern is contrary to the common belief that slower heating can be used to allow acclimation and consequently higher tolerance, although it is consistent with several recent studies on aquatic ectotherms (Illing et al., 2020; Saravia et al., 2021). This is best explained by the fact that slow heating allows more time for cumulative heat damage, such as protein denaturation, oxidative stress, and metabolic exhaustion, to occur, causing the crab to lose its righting response at a lower temperature. Conversely, rapid heating can outrun the accumulation of the damage, resulting in a higher endpoint temperature (Terblanche et al., 2011). The practical impact is that CTMax values measured at unnaturally rapid heating rates (typical in the literature) may indicate exaggerated thermal tolerance in the case of real-world, slowly developing heat waves. It suggests that future researchers should consider ramping rates that are representative of the field conditions (e.g. 1°C h^−1^ or slower).

It was found that moderate warming (31°C) can increase metabolism and reduce activity, whereas extreme warming (33°C) can lead to behavioral shutdown and switching to protein catabolism. These effects happen at temperatures only 4–6°C above present temperatures, meaning that crabs become ecologically nonfunctional before reaching lethal levels (CTMax) of about 33–35.5°C. This would have a great effect on the population growth since there would be less energy consumption and reproduction.

Under the Representative Concentration Pathway (RCP8.5) scenario proposed by the Intergovernmental Panel on Climate Change (IPCC), with an average temperature of 31°C projected by 2100 (+4°C), most of the streams in Northern Pakistan will reach temperatures above 31°C, driving *H. emphyseteum* into burrows. Thermal refugia and acclimation will sustain its survival, but due to limited dispersal and fragmented habitats, range shifts will be challenging. Riparian vegetation and hydrological connectivity should be conserved.

The lack of natural temperature and predation variation and a limited sample size of physiological variables are the limitations. The CTMax ramping rates were not realistic to the ecological context, and there was no measurement of the long-term fitness outcomes. Additionally, exclusive use of adult males limits the generalizability of our findings to females and juveniles. Future studies should investigate sex-specific and ontogenetic differences in thermal responses to fully assess population-level vulnerability. Future studies should consider chronic exposure, include both sexes and look at multi-stressor situations, complemented by molecular analyses to identify the underlying mechanisms.

### Conclusion

Sublethal effects in *H. emphyseteum* occur at warming levels of 4–6°C above ambient temperatures before reaching lethal levels. These involve heightened metabolism, catabolism of proteins and termination of surface activity and foraging. Burrow retreat is a short-term mechanism but reduces energy consumption and mating. Thermal tolerance is not static, and its warming rate depends on how much it warms, and *H. emphyseteum* is most likely to be severely challenged by moderate warming. To curb the effects of climate change, conservation should be directed toward protecting riparian shading and stream flow.

## MATERIALS AND METHODS

### Specimen collection and acclimation

Adult male *H. emphyseteum*, with a carapace width of 32.29–37.74 cm and a wet weight of 12.5–17.3 gm (*n*=36 for physiology and behavior; *n*=30 for CTMax), were collected from a rocky lodge at Ghar Uba, Landi Kotal, Khyber District, Pakistan (33°57′ N, 71°24′ E). This area has a mean water temperature of approximately 29±0.5°C. Adult male crabs were exclusively used in this study to control potential sex-related differences in physiology, behavior, and energy allocation, particularly as females may carry eggs or allocate energy to reproduction, which could confound thermal responses. This study therefore provides a baseline assessment of male thermal physiology. Crabs were transported in plastic containers to the Institute of Zoological Sciences, University of Peshawar, and only healthy crabs with no visible physical injury were selected for the trial. These crabs were acclimated for 7 days in individual compartments of aerated 72 l aquaria (six compartments per aquarium) within a closed recirculating system under controlled conditions [29±0.5°C, dissolved oxygen (DO) 95–100% saturation]. Water quality parameters (temperature, DO, pH, ammonia, nitrite, nitrate) were monitored daily using a multiparameter water quality meter (YSI ProDSS). Ammonia and nitrite levels were maintained below 0.1 and 0.5 mg l^−1^, respectively, through regular partial water changes (20% daily). Each aquarium was equipped with a closed recirculating system incorporating mechanical, biological (bio-balls with nitrifying bacteria), and activated carbon filtration. Crabs were fed *ad libitum* with commercial sinking pellets for freshwater crustaceans (Tetra Crustacean Mini, Tetra GmbH, Germany; protein content 42%, fat 6%) and were starved for 24 h before experimentation to minimize handling stress.

### Experimental design

In this study, a total of 66 crabs were used. These were divided into two separate experimental groups: 30 crabs were kept solely for determining their CTMax, and 36 crabs were used for the main physiological and behavioral experiments. In the main experiment, the 36 crabs were randomly distributed among three temperature treatments (12 crabs per treatment), corresponding to the current water temperature of Khyber Pakhtunkhwa according to the Pakistan Meteorological Department and projected increases of +4°C (31°C) and +6°C (33°C) by the end of the century (IPCC, 2014).

The target temperature was achieved by gradually increasing the water temperature from 27°C (acclimation temperature) at a rate of 1°C day^−1^. Crabs were then held at their respective treatment temperatures for 7 days prior to physiological and behavioral measurements to allow for thermal acclimation (following the recommendations of Stillman, 2003). Twelve crabs were housed individually in separate compartments within 72 L aquaria (six compartments per aquarium, with two replicate aquaria per temperature), enabling individual identification while maintaining consistent environmental conditions. The use of replicate aquaria per treatment minimized the potential for pseudo-replication and allowed tank effects to be assessed. The same individuals were used for physiological measurements and behavioral observations, with behavioral recordings conducted on day 6 of the temperature treatments, followed by physiological measurements on day 7. This sequence ensured that behavioral responses were assessed without prior handling stress from physiological measurements. Additionally, for the determination of CTMax, a total of 30 crabs (ten per heating rate) were utilized.

### Oxygen consumption and ammonia excretion

Oxygen consumption and ammonia excretion were measured on a single crab per respirometry chamber at a time, with measurements conducted in cohorts of four crabs per day (one from each temperature treatment: 27, 31, and 33°C, plus a chamber blank for background correction), balanced across temperature treatments and replicated over 3 days to account for potential day effects. All 12 individuals per temperature treatment were measured across three separate days (four crabs per day) to avoid potential temporal batch effects. OCR was measured in airtight cylindrical glass respirometry chambers (10 cm diameter×15 cm height; 1.18 l volume, filled with 1.0 l water) equipped with magnetic stirrers to ensure homogeneous oxygen distribution and prevent stratification. DO was measured using a calibrated fiber-optic DO meter (FireStingO_2_, PyroScience GmbH, Germany) with a precision of ±0.1 mg O_2_ l^−1^. Water samples for DO and ammonia excretion measurement were gently withdrawn from the chamber's center using a syringe connected to a sampling port, minimizing disturbance to the crab. To avoid oxygen gradients, chambers were continuously stirred at a low speed that did not disturb the crabs. Initial DO (DO_0_) was measured after a 30-min acclimation period, then chambers were sealed for 2 h, and the final DO (DO_f_) was measured. DO_0_ was constant at 8.2 mg l^−1^ (tap water). OCR was calculated as follows:

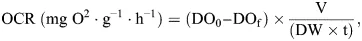
where V=1 L, DW=crab dry weight (0.2 g), and t=2 h. After completion, crabs were oven dried at 60°C for 48 h to measure dry weight.

Ammonia concentration in water samples was determined using the indophenol blue method with UV-visible spectrophotometry (Shimadzu UV-1800, Japan) at 640 nm wavelength. Standard curves were prepared using ammonium chloride solutions (0.05–1.0 mg NH_4_^+^ L^−1^; *R*^2^>0.999). Water samples were filtered through 0.45 μm syringe filters prior to analysis to remove particulate matter. All samples were analyzed in triplicate, and results were blank corrected using control water samples (Solorzano, 1969). AER was calculated as follows:

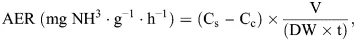
where C_s_ represents the concentration of the sample, and C_c_ represents the concentration of the control, which was equal to 5.0 μg L^−1^, V=1 L, DW=0.2 g, and t=2 h.

Q_10_ was used to quantify the thermal sensitivity of OCR across the experimental temperature range. Although Q_10_ traditionally describes the change in a physiological or metabolic rate associated with a 10°C increase in temperature, it can also be calculated from observations obtained at any two temperatures by extrapolating the observed rate difference to a 10°C interval. This approach has been applied in crustacean studies to assess the temperature dependence of metabolic processes over smaller temperature increments.

Q_10_ for oxygen consumption was calculated according to the following equation:

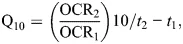
where OCR_1_ and OCR_2_ represent the mean OCRs measured at temperatures *t*_1_ and *t*_2_, respectively. Q_10_ values were calculated using mean oxygen consumption data for the temperature intervals 27–31 and 27–33°C. A Q_10_ value of 1 indicates that oxygen consumption is independent of temperature, whereas values greater than 1 indicate an increase in oxygen consumption with increasing temperature. Thus, Q_10_ provides an index of the thermal sensitivity of the metabolic rate and can be used to evaluate the physiological response of the study organism to temperature variation.

### Behavioral observations

Behavioral responses were assessed in microcosms simulating natural habitat conditions. The microcosms consist of glass aquaria (50 cm×30 cm×30 cm; water column height: 15 cm) with a 10 cm sediment layer (fine sand and gravel, <2 mm diameter). The sediment temperature was monitored at 5 cm depth using a soil temperature probe; it remained within ±0.3°C of the water temperature due to the shallow water column and uniform heating system. Thus, crabs could not use burrows as thermal refugia within the experimental setup. Crabs were allowed to construct their own burrows during a 48-h acclimation period prior to behavioral recordings. Each aquarium was maintained at one of the experimental temperatures (27, 31, or 33°C) under a 12-h light:12-h dark photoperiod. After 48 h of exposure, 60-min video recordings were made. The following three behaviors were quantified: feeding rate, which is the number of claw-to-mouth feeding events per minute; surface activity, which refers to the total time (min h^−1^) the crab was actively moving, foraging, or walking on the sediment surface; and burrow retreat, which is the total time (min h^−1^) the crab remained fully inside its burrow with only the chelipeds or eyes occasionally visible.

Time not accounted for in these categories represents periods when crabs partially emerged from burrows but were not fully active on the surface (e.g. burrow maintenance or stationary at burrow entrance), as well as brief periods when individuals were not visible due to angles or temporary movement behind structures.

Crabs were fasted for 24 h prior to the observation period. At the start of each observation session, a standardized amount of commercial food (0.5 g of sinking pellets per crab; Tetra Crustacean Mini) was placed in the center of each aquarium. Behavior was recorded using high-definition video cameras for 60 min starting immediately after food addition. Behaviors were scored by two observers who were blind to treatment assignment. Inter-observer reliability was assessed using Cohen's kappa coefficient (with κ=0.89 for feeding events, κ=0.91 for surface activity, and κ=0.87 for burrow retreat, indicating an excellent agreement).

### CTMax

CTMax was measured following controlled dynamic heating protocols adapted from Mora and Ospina (2001). Individual crabs were placed in a thermostatically regulated water bath (Grant Instruments, Cambridge, UK) with continuous aeration. Water temperature was measured continuously using a calibrated thermocouple probe (±0.1°C accuracy) positioned adjacent to the crab (within 1 cm) to ensure accurate measurement of the crab's immediate thermal environment. Temperature was increased from the acclimation temperature (29°C) at three independent ramping rates: 1°C min^−1^, 1°C per 30 min, and 1°C h^−1^ (*n*=10 per rate, total *n*=30). The temperature controller's accuracy was verified against a certified mercury thermometer prior to each trial. For the 1°C min^−1^ treatment, temperature was increased using the water bath's pre-programmed heating function; for slower ramping rates, temperature was increased using programmable temperature controllers (PID controllers with 0.1°C precision).

The CTMax endpoint was defined as the temperature at which a crab exhibited loss of righting response, identified by failure to return to an upright position within 10 s after gentle tactile stimulation of the walking legs. Upon reaching CTMax, the crabs were immediately transferred to recovery tanks at the acclimation temperature. All crabs recovered fully within 30 min of transfer.

### Statistical analysis

All analyses were performed using IBM SPSS 27.0.1. Data distributions were assessed using the Shapiro–Wilk test and the homogeneity of variances was evaluated using Levene's test. Temperature effects on individual physiological and behavioral variables were assessed using one-way ANOVA when the assumption of homogeneity of variance was satisfied. Welch's one-way ANOVA was used for variables showing significant heterogeneity of variance. Tukey's HSD was used for post hoc pairwise comparisons following conventional ANOVA, whereas Games–Howell comparisons were used following Welch's ANOVA. For CTMax, one-way ANOVA followed by Tukey's HSD was used to compare the three heating-rate treatments. Q_10_ values were calculated for OCR between 27–31 and 27–33°C, with 95% CIs obtained from 5000 bootstrap resamples. The relationship between metabolic rate (OCR) and behavior was assessed using bivariate Pearson's correlations, conducted independently for each experimental temperature to determine if the strength of the physiological–behavioral linkage was temperature dependent. Statistical significance was accepted at *P*<0.05.

## Supplementary Material



10.1242/biolopen.062895_sup1 Supplementary information
