# Supplementary material for: Ecophysiology and behavioral indicators of thermal stress in freshwater crab Himalayapotamon emphyseteum under experimental warming
Source: Biol Open. 2026 Oct 21;15(10):bio062895. doi: 10.1242/bio.062895 (PMC13730130; doi:10.1242/bio.062895)
Supplement: Supplementary file 1 [file biolopen-15-062895-s1.pdf]

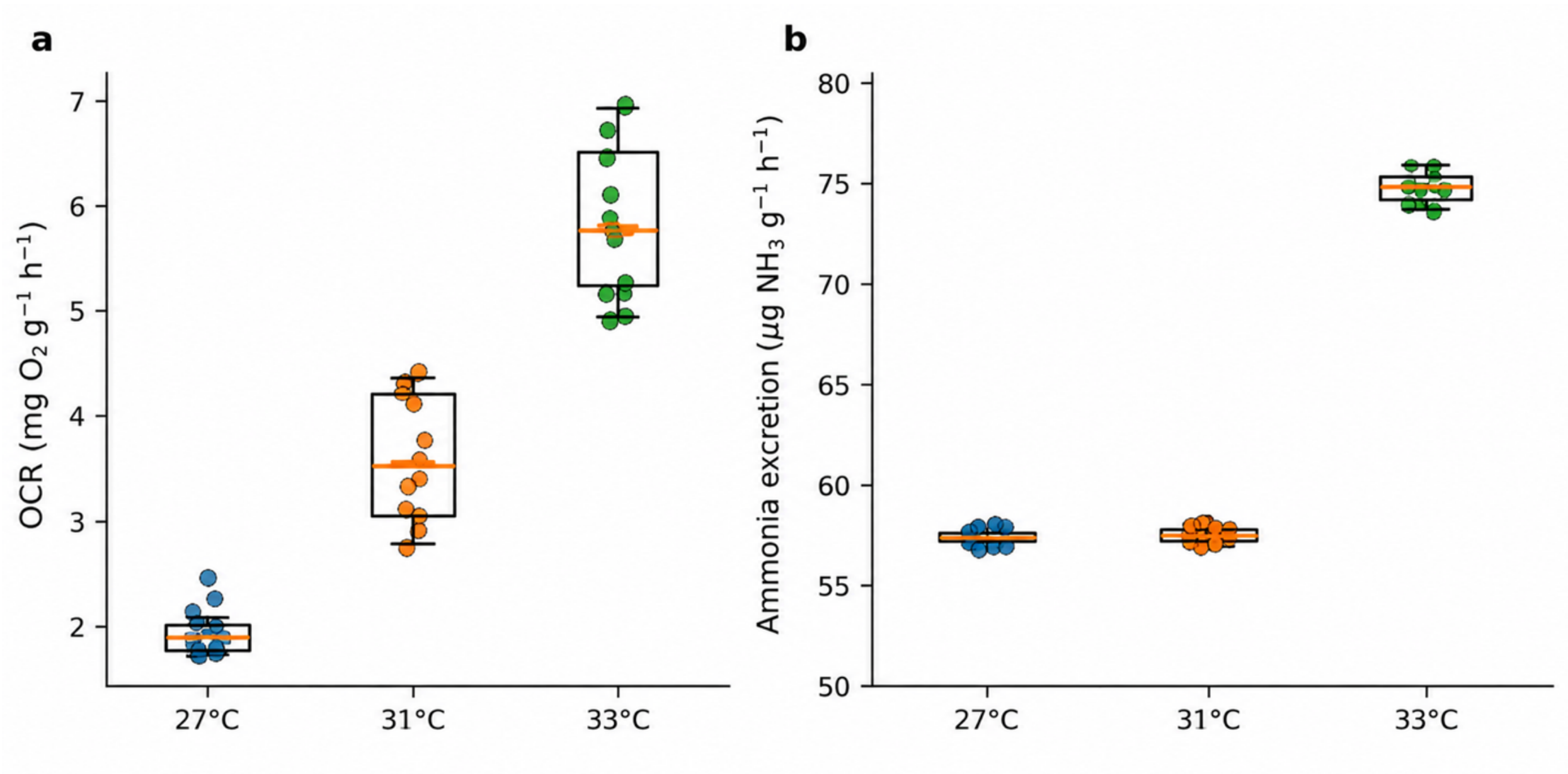

Fig. S1. Physiological responses of H. emphyseteum . to temperature. (a) Oxygen consumption rate and (b) ammonia excretion rate at 27, 31 and 33°C. Individual observations are shown as points; boxes indicate the interquartile range, horizontal lines indicate medians, and thick horizontal marks indicate group means. n=12 per temperature.

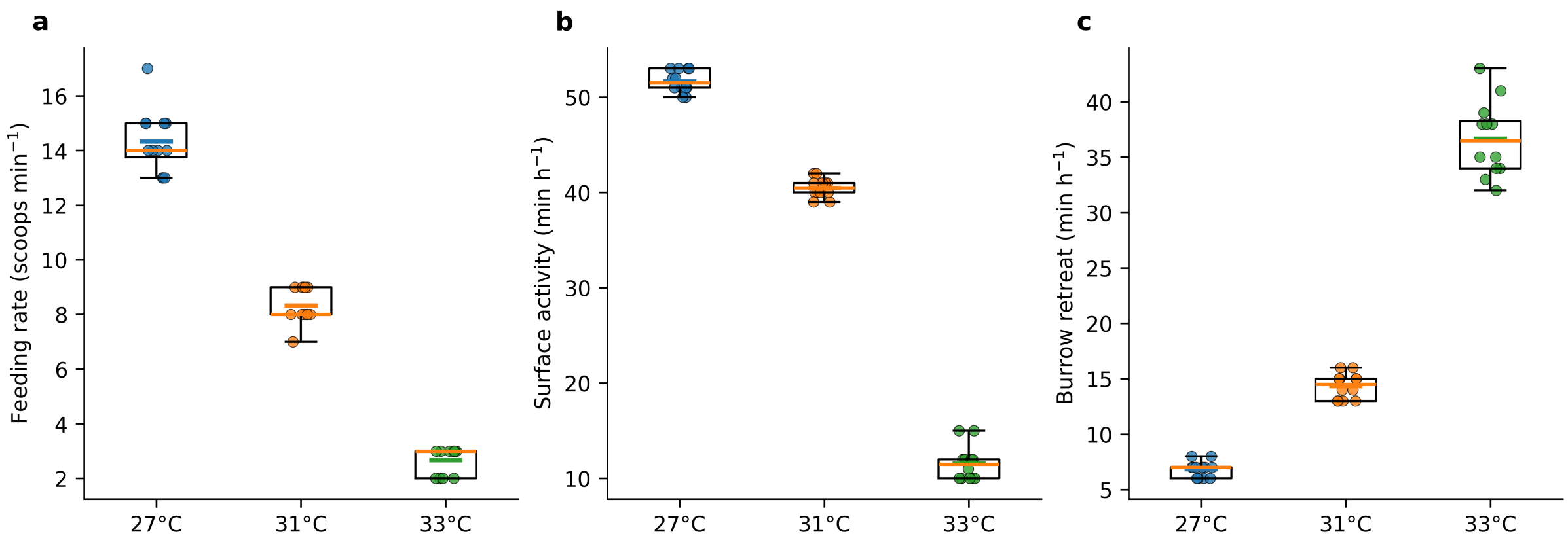

Fig. S2. Behavioural responses of H. emphyseteum to temperature. (a) Feeding rate, (b) surface activity and (c) burrow retreat at 27, 31 and 33°C. Individual observations are shown as points; boxes indicate the interquartile range, horizontal lines indicate medians, and thick horizontal marks indicate group means. n=12 per temperature. All pairwise temperature comparisons were significant (Tukey's HSD, P<0.001).

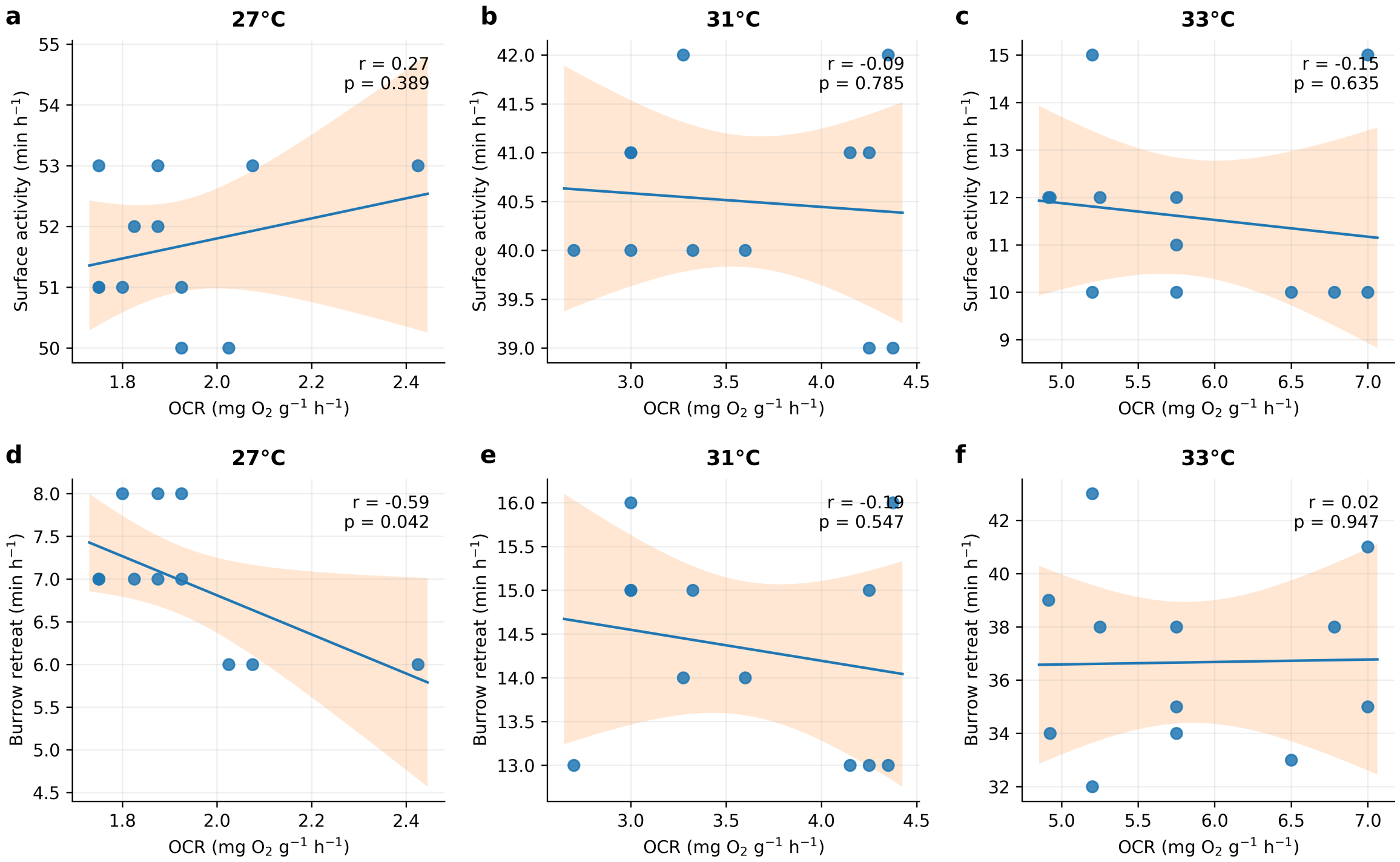

Fig. S3. Relationships between oxygen consumption rate (OCR) and behavioural responses at 27, 31, and 33°C. Panels (a–c) show the relationships between OCR and surface activity, and panels (d–f) show the relationships between OCR and burrow retreat. Points represent individual crabs, solid lines represent linear regression fits, and shaded bands indicate 95% confidence intervals. Pearson's correlation coefficient (r) and associated p-value are shown in each panel.

Biology Open • Supplementary information

Table S1. Values are mean ± SD; n = 12 independent crabs per temperature treatment. Different superscript letters within a row indicate significant differences among temperature treatments. Tukey's HSD was used for ammonia excretion, feeding rate and surface activity; Games–Howell comparisons were used for OCR and burrow retreat because of unequal variances.

**Parameter**	**27°C (n=12)**	**31°C (n=12)**	**33°C (n=12)**
Oxygen consumption rate (mg O_2 g^{-1} h^{-1})	1.917 ± 0.192[a]	3.606 ± 0.631[b]	5.835 ± 0.792[c]
Ammonia excretion rate (µg NH_3 g^{-1} h^{-1})	57.568 ± 0.013[a]	57.582 ± 0.049[a]	75.088 ± 0.042[b]
Feeding rate (claw-to-mouth- feeding events min^{-1})	14.33 ± 1.15[a]	8.33 ± 0.65[b]	2.67 ± 0.49[c]
Surface activity (min h^{-1})	51.67 ± 1.15[a]	40.50 ± 1.00[b]	11.58 ± 1.83[c]
Burrow retreat (min h^{-1})	6.83 ± 0.72[a]	14.33 ± 1.15[b]	36.67 ± 3.37[c]

Table S2. Q_{10} values were calculated from Oxygen Consumption rate between 27-31°C and 27–33°C . The 95% confidence intervals were estimated by bootstrap resampling of the individual OCR observations.

S.NO	Temperature interval	Value	Value 95% CI
1	Q_{10} (27–31°C)	4.86	(3.67–6.29)
2	Q_{10} (27–33°C)	6.40	(5.48–7.40)

Table S3. Values are mean ± SD. Different superscript letters indicate significant differences among heating rates according to Tukey's HSD test ($p < 0.05$). One-way ANOVA: $F_{2,27} = 30.50$, $p < 0.001$, $\eta^2 = 0.693$. Ten independent individuals were tested at each heating rate.

**S.NO**	**Heating rate**	**CTMax (°C)**
1	1°C min^{-1} (n = 10)	35.60 ± 0.70[a]
2	1°C per 30 min (n = 10)	34.10 ± 0.74[b]
3	1°C h^{-1} (n = 10)	32.90 ± 0.88[c]
