# Supplementary material for: Ecophysiology and behavioral indicators of thermal stress in freshwater crab Himalayapotamon emphyseteum under experimental warming
Source: Biol Open. 2026 Oct 21;15(10):bio062895. doi: 10.1242/bio.062895 (PMC13730130; doi:10.1242/bio.062895)
Supplement: Supplementary file 2 [file biolopen-15-062895_review_history.pdf]

Ecophysiology and Behavioral Indicators of Thermal Stress in Freswater Crab Himalayapotamon emphyseteum Under Experimental Warming

Tanzeela Tanzeela, Muhammad Adnan, Sanaullah Khan and Aisha Gul
10.1242/bio.062895

Editor: Valentina di Santo

Review timeline

Original submission:	22 August 2026
Editorial decision:	1 September 2026
First revision received:	9 September 2026
Accepted:	14 September 2026

Original submission

First decision letter

MS ID#: bio.062895

MS Title: Ecophysiology and Behavioral Indicators of Thermal Stress in Freswater Crab Himalayapotamon emphyseteum Under Experimental Warming

Authors: Tanzeela Tanzeela, Muhammad Adnan, Sanaullah Khan and Aisha Gul

I have now reached a decision on the above manuscript.

The reviewer reports are shown at the bottom of this email.

As you will see, the reviewers raised a number of substantial criticisms that prevent me from accepting the paper at this stage.

They suggest, however, that a revised version might prove acceptable, if you can address their concerns. If you think that you can deal satisfactorily with the criticisms on revision, I would be pleased to see a revised manuscript. We would then return it to the reviewers.

At this stage, we also ask you to ensure your manuscript complies with our formatting guidelines. Provided you are able to fully address the referees' comments, we are positive about publication of your paper (we accept over 95% of revision submissions) and therefore hope you won't mind any extra work involved in reformatting your manuscript at this point.

Please upload both a 'clean' version of your Word file, along with a highlighted version clearly showing where you have made changes in the revised manuscript. Please avoid using 'Track changes' in Word files as these are lost in PDF conversion.

I should be grateful if you would also provide a point-by-point response detailing how you have dealt with the points raised by the reviewers in the 'Response to Reviewers' box. Please attend to all of the reviewers' comments. If you do not agree with any of their criticisms or suggestions please explain clearly why this is so.

© 2026. Published by The Company of Biologists under the terms of the Creative Commons Attribution License (https://creativecommons.org/licenses/by/4.0/).

Reviewer 1

Comments for the author

The manuscript describes a study on temperature effects on some physiological and behavioural measures of a freshwater crab. The data are potentially useful from a climate change perspective and this is as far as I can tell the first such study made on this species.
However, the manuscript is far from a state that could be published. Throughout grammar and spelling needs to be improved. Here are some more specific comments:
Title: Few people will know what type of animal this is from seeing the Latin name so please add "the freshwater crab" in front of the Latin name in the title.

Page. 2, line 23-38: Same sentence repeated.

Page 3, line 25: change "measured as a measure" to "used as a measure"

Page 4, line 5: Why "Himalayapotamon sp." here (and also elsewhere in the text) when you apparently know he species? Should also be in italics. Also change this in the figure legends and text. Moreover, change "from rocky streams" to "from a rocky stream" since the methods section indicate that they were collected from a single site.

Page 4. Line 52-53: Define "scopes" in the material section or write out "number of claw-to-mouth feeding events" if that is what it means.
Throughout the ms (text, table and figure) I suggest you change the unit of ammonia excretion from mg NH_3 g^{-1} h^{-1} to to µg NH_3 g^{-1} h^{-1} to avoid all those zeros.

Materials: In general this section is much too rudimentary to allow anyone to repeat this study. It needs to be much more detailed. Here are some examples:
Collection and acclimation: What was the size (weight) of the crabs and how many per aquarium? Was the water quality of the closed recirculation system monitored? Was there some biological filter in the system to maintain water quality? Is there "commercial food" for crabs and what is it?
Microcosms: What was the measures of the aquaria and how high was the water column? Was the temperature in the sediment measured and was it the same as in the water? It is important to know if the crabs could cool down in the burrows. What type of sediment was used and did the crabs make their own burrows? Was only one crab at the time put in the aquaria?
Oxygen consumption: Describe the chambers (circular or box, measures) and how water got in and out of them. What method and equipment were used to measure oxygen (electrodes?). How was the water sampled from the chambers to avoid an influence of oxygen gradients in the water.
Ammonia: Give a reference to the method used. To only state "UV-visible spectrometry" is clearly not enough to explain how it was done.
Behavioural observations: What does surface activity mean? Was it just that they were present at the surface? In that case the sum of burrow retreat and surface activity should be 60 min / h but that is not the case (Table 1). Please explain this in the methods.
CTmax: In the table and figure n=10 is stated while in the methods n=8 is stated. What was it?

Discussion:
I find the whole paragraph on OCR very confusing and it needs to be rewritten. Here are some examples:
Page 8, line 18-19. What does "i-e Cohen 5.87" mean. What does "Q10 values (3.18 4.06)" mean? The Q10 values should be given in the results section. The statement "supports the biological scale of this hike" is not easy to understand.
Page 8, line 33. "Hence, the decrease OCR at 33C can be considered as thermal limitation" does not make sense to me since OCR actually increased from 31 to 33 °C.

Other issues in the Discussion:
Page 9, line 31. should be "increased by six times".
Page 10, line 9. Explain how a long-lasting retreat into burrows may elevate the vulnerability of the H emphyseteum to predators. Are not the burrows a protection against predators?

© 2026. Published by The Company of Biologists under the terms of the Creative Commons Attribution License (https://creativecommons.org/licenses/by/4.0/).

Page 10, line 36. "do not become ecologically non-functional". I assume you mean the opposite.
Page 12. The text under the headings Limitations and Conclusion should be moved to the end of the Discussion.

Reviewer 2

Comments for the author

This manuscript examines physiological and behavioural responses of the freshwater crab Himalayapotamon emphyseteum to experimental warming, including oxygen consumption, ammonia excretion, feeding and activity, burrow retreat, and CTMax measured using different heating rates. I found the Introduction particularly strong. It is clearly written, provides a good rationale for considering multiple physiological and behavioural responses, and does a good job of explaining why sublethal responses to warming may be ecologically important. The combination of physiological and behavioural measurements is also potentially valuable, particularly given the relative lack of information for freshwater crabs from this region.

However, I have several substantial concerns with the experimental description and interpretation of the results. Most importantly, considerable methodological information is currently missing, making it difficult to evaluate the experimental design and statistical analyses, or to reproduce several components of the study. There are also some important issues with the interpretation of oxygen consumption as a thermal performance curve. I think these issues need to be addressed before the the conclusions can be properly assessed.

1. The Introduction is very well written and clearly establishes the rationale for all of the measurements made in the study. However, it would be strengthened by ending with explicit hypotheses or predictions. At present, the final paragraph simply states that the authors measure oxygen consumption, ammonia excretion, Q10, CTMax and several behaviours, which makes the study appear largely observational. There are clear predictions that could be made from the preceding literature, for example that warming should increase metabolic demand, alter nitrogen excretion, suppress feeding and surface activity, increase refuge use, and that measured CTMax may depend on heating rate.

2. The experiment appears to use adult males exclusively. This needs justification, especially given the broad conclusions being drawn about the thermal physiology and vulnerability of the species, excluding females is potentially a major limitation, particularly if sex differences in behaviour, energetic demand or thermal responses are possible.

3. Section 4.2 currently provides very little information beyond the temperatures and the number of individuals assigned to each treatment. It is not clear whether these represent acute temperature exposures or acclimation treatments. If the animals were acclimated to 27, 31, or 33°C, how were they brought to these temperatures, how long were they held there before measurements, and how many independent holding tanks were used per treatment? If all crabs within a treatment were maintained in a single tank, then tank effects and non-independence become an important concern.

4. Section 4.3 is currently too brief to allow the oxygen consumption measurements to be properly evaluated. The authors state that crabs were placed into 1-L closed chambers, allowed 30 min before initial oxygen measurement, and then sealed for two hours. Several important details are missing. How many crabs were measured simultaneously? Were individuals measured in cohorts, and if so, was cohort balanced across temperatures? Were all 12 animals from a particular temperature measured on the same day, or across multiple days? These factors could potentially introduce batch effects that should be included in the statistical models. It is also not stated how background microbial oxygen consumption was measured and corrected for.

In addition, a 30-min period following transfer to a chamber may be insufficient for handling-induced metabolic elevation to decline. With only a single two-hour closed measurement, it is difficult to distinguish temperature effects on routine metabolism from differences among temperatures in recovery from handling and transfer. The authors should discuss this issue and, if possible, provide evidence that oxygen uptake had stabilised before measurements began. Final

© 2026. Published by The Company of Biologists under the terms of the Creative Commons Attribution License (https://creativecommons.org/licenses/by/4.0/).

oxygen concentrations in the chambers should also be reported so that readers can establish whether oxygen declined sufficiently during the two-hour period to itself influence metabolic rate (i.e. whether or not hypoxia occurred in the chambers and whether this varied across temperatures).

5. There is also a potential confound within these closed respirometry trials. Ammonia excretion increased substantially at 33°C, while oxygen consumption was simultaneously highest at this temperature. The authors calculate ammonia excretion from the same two-hour incubation period used for the physiological measurements. If ammonia accumulated within the sealed chambers during these trials, animals in the warmest treatment may therefore have experienced both the highest temperature and the highest ammonia exposure. Depending on the concentrations reached, this could itself influence physiology and oxygen uptake. The authors should report the actual final ammonia concentrations and consider whether ammonia accumulation could have affected the measurements.

6. Section 4.4 is also missing substantial methodological information. The manuscript states that crabs were housed in 19-L aquaria, exposed for 48 h, and subsequently recorded for 60 min. However, it is not clear whether animals were housed individually or in groups, how many aquaria were used per temperature, how animals were allocated among tanks, or whether the same individuals were subsequently used for physiological measurements.
The feeding measurement particularly needs clarification. What food was provided, how much was provided, and when relative to the observation period? Were animals previously fasted? Were all behavioural measurements taken during a feeding trial, or was feeding assessed during a separate observation period? It is also important to specify whether behaviours were scored live or from video recordings, whether observers were blind to treatment, and whether repeatability among observers was assessed if multiple observers were involved. These details are necessary both for replication and for evaluating whether tank identity or observation cohort should be included in the statistical analyses.

7. Section 4.5 states that animals were placed in a thermostatically regulated water bath and subjected to three ramping rates, but more information is needed concerning how those rates were achieved. How was temperature measured during each trial, where was the temperature probe located relative to the animal, how accurately was temperature controlled, and what equipment was used to produce the specified heating rates? This is especially important for the 1°C min^{-1} treatment, where even relatively small delays between water temperature and animal body temperature could influence the apparent CTMax. There also appears to be an inconsistency in sample size. The Methods state that 24 individuals were used for CTMax, with eight per heating rate, whereas the Results report ten individuals per heating rate.

8. I can't evaluate the statistical analyses because the experimental structure is unclear. The analyses treat individual crabs as independent observations and primarily use one-way ANOVAs. However, whether this is appropriate depends on how animals were housed and measured. If multiple crabs shared holding tanks, behavioural aquaria or respirometry cohorts, these sources of non-independence may need to be incorporated as random effects. The authors therefore need to fully describe the experimental structure before the statistical approach can be assessed.

9. It would also be useful to clarify whether oxygen consumption/ammonia excretion and behavioural measurements were made on the same individuals. If so, there is potentially a interesting analysis available here that is not currently explored. For example, do individuals with higher oxygen consumption show greater reductions in feeding or activity, or greater burrow use? More interestingly, does the relationship between physiology and behaviour itself change with temperature? Examining individual-level correlations, potentially using temperature × physiological-trait interactions, could provide much stronger evidence for the proposed coordination between metabolic and behavioural responses than simply showing that the mean of each trait changes with temperature.

10. I don't think the interpretation of oxygen consumption in the Discussion is currently justified. The manuscript states: "H. emphyseteum did not experience metabolic depression up to 33C, hence the thermal performance curve … is no longer hump-shaped." However, the data show oxygen consumption increasing across the three temperatures tested, with the highest value at 33°C. This

© 2026. Published by The Company of Biologists under the terms of the Creative Commons Attribution License (https://creativecommons.org/licenses/by/4.0/).

does not demonstrate that the complete thermal performance curve is not hump-shaped. Higher experimental temperatures may simply have revealed the descending portion of the curve.

In addition, the classic hump-shaped performance curve being discussed here is normally considered in relation to organismal performance or aerobic scope, whereas the current study measures oxygen consumption alone. Increasing resting or routine oxygen uptake with warming does not necessarily indicate increasing capacity for aerobic performance.

A related concern applies to the statement that a Topt of 35°C was estimated from a quadratic model. I can't find this quadratic model described in the Methods or Results. More importantly, with only three oxygen-consumption temperatures, all showing a steep increase, I don't see how a biologically meaningful optimum can really be extrapolated.

It's also not clear why the temperature associated with the highest rate of resting or routine oxygen consumption should be called an "optimum". High metabolic expenditure may instead indicate high maintenance costs, and aerobic scope could conceivably be most constrained at that temperature. I would strongly recommend removing this estimated Topt unless the authors can provide a much stronger methodological and conceptual justification.

11. Also in the discussion, the authors state that "the decrease OCR at 33C can be considered as thermal limitation", yet oxygen consumption actually increased substantially from 31 to 33°C and was highest at 33°C. It is therefore unclear what decrease is being referred to. The subsequent interpretation in terms of oxygen- and capacity-limited thermal tolerance also requires revision. OCLTT concerns limitations to aerobic capacity/aerobic scope as temperatures approach thermal limits. Resting or routine oxygen uptake increasing with temperature, on its own, cannot demonstrate oxygen limitation. No maximum metabolic rate or aerobic scope was measured in this study.

12. To me, one of the most interesting findings in the manuscript receives surprisingly little discussion: metabolic expenditure increases dramatically with temperature at exactly the same time that feeding declines. Oxygen consumption at 33°C is approximately three times that at 27°C, while feeding is reduced by around 80%. This creates a potentially important energetic mismatch, with increasing maintenance costs occurring while energy acquisition is simultaneously suppressed. That may be considerably more ecologically meaningful than trying to infer a metabolic optimum from the oxygen-consumption data. It could provide a strong mechanistic link between the physiological and behavioural components of the study and potentially explain why substantial ecological effects could occur well below CTMax. I would encourage the authors to make much more of this result in the Discussion.

Reviewer's Responses to Questions

Experimental quality
Are experiments performed using appropriate methods that will answer the question (or test the hypothesis or support the observations) posed by the authors? Is the right tool used for the job?
If 'No', please indicate reasons in Comments for Author box below.
Reviewer #1:
- Yes

Reviewer #2:
- Yes

Were the data analyzed using appropriate statistical tests?
If 'No', please indicate reasons in Comments for Author box below.
Reviewer #1:
- Yes

Reviewer #2:
- No

© 2026. Published by The Company of Biologists under the terms of the Creative Commons Attribution License (https://creativecommons.org/licenses/by/4.0/).

Reproducibility
Were experiments performed using adequate number of biological replicates?
If 'No', please indicate reasons in Comments for Author box below.
Reviewer #1:

- Yes

Reviewer #2:

- Yes

Is there sufficient raw data to assess the rigor of the analysis?
If 'No', please indicate reasons in Comments for Author box below.
Reviewer #1:

- Yes

Reviewer #2:

- Yes

Does the methods section provide sufficient detail to permit reproducibility?
If 'No', please indicate reasons in Comments for Author box below.
Reviewer #1:

- No

Reviewer #2:

- No

Completeness
Are the manuscript's conclusions supported by the data?
If 'No', please indicate reasons in Comments for Author box below.
Reviewer #1:

- Yes

Reviewer #2:

- No

Scholarship
Do the authors cite and discuss the merits of data that would argue for and against their conclusion?
If 'No', please indicate reasons in Comments for Author box below.
Reviewer #1:

- Yes

Reviewer #2:

- Yes

Does the manuscript title & abstract accurately reflect the contents of the manuscript, without hyperbole?
If 'No', please indicate reasons in Comments for Author box below.
Reviewer #1:

- Yes

Reviewer #2:

- Yes

© 2026. Published by The Company of Biologists under the terms of the Creative Commons Attribution License (https://creativecommons.org/licenses/by/4.0/).

First revision

Author response to reviewers' comments

Dear Editor,

We are thankful to the reviewers of the manuscripts for their thorough and constructive comments. We have carefully considered their comments and incorporated in the manuscript. Below, we provide a detailed point-by- point response to each comment. All these incorporated changes have been highlighted in green (for reviewer's 1 comments) and in yellow for (reviewer's 2) comments so that they are easy to read.

RESPONSE TO REVIEWER 1

Reviewer 1's General Comment: The manuscript describes a study on temperature effects on some physiological and behavioural measures of a freshwater crab. The data are potentially useful from a climate change perspective and this is as far as I can tell the first such study made on this species. However, the manuscript is far from a state that could be published. Throughout grammar and spelling needs to be improved.

Response: We sincerely thank the reviewer for recognizing the novelty and potential of this study. We have thoroughly revised and edited the manuscript for grammar, spelling, and clarity.

1.Title: Few people will know what type of animal this is from seeing the Latin name so please add "the freshwater crab" in front of the Latin name in the title.

Response: We agree with this important suggestion and revised the title to: "Ecophysiology and Behavioral Indicators of Thermal Stress in the Freshwater Crab *Himalayapotamon emphyseteum* Under Experimental Warming"

2. Page 2, line 23-38: Same sentence repeated.

Response: We apologize for this error. The duplicate sentence has been removed from the abstract. The abstract now reads concisely without any repetition.

3.Page 3, line 25: Change "measured as a measure" to "used as a measure".

Response: The sentence now reads: "Oxygen uptake is typically used as a measure of metabolic rate and aerobic capacity..." (Introduction, paragraph 2).

4. Page 4, line 5: Why "Himalayapotamon sp." here (and also elsewhere in the text) when you apparently know the species? Should also be in italics. Also change this in the figure legends and text. Moreover, change "from rocky streams" to "from a rocky stream" since the methods section indicate that they were collected from a single site.

Response: We appreciate these corrections. We have changed all instances of Himalayapotamon sp. to *Himalayapotamon emphyseteum* (italicized throughout). Corrected "from rocky streams" to "from a rocky stream" (Introduction, final paragraph). Updated figure legends to reflect the full species name in italics

5. Page 4, Line 52-53: Define "scopes" in the material section or write out "number of claw-to-mouth feeding events" if that is what it means.

Response: We agree and have replaced this term throughout the manuscript as suggested by the

© 2026. Published by The Company of Biologists under the terms of the Creative Commons Attribution License (https://creativecommons.org/licenses/by/4.0/).

reviewer for clarity. The Methods section (Section 4.4) now explicitly defines Feeding rate: number of claw-to-mouth feeding events per minute.

6. Throughout the manuscript: I Suggest changing the unit of ammonia excretion from mg NH_3 g^{-1} h^{-1} to µg NH_3 g^{-1} h^{-1} to avoid all those zeros.
Response: Excellent suggestion. We have converted all ammonia excretion values from mg to µg NH_3 g^{-1} h^{-1} throughout the manuscript, including Abstract, Results section and Table 1, Figure 1b and methods section. All values and statistical analyses have been updated accordingly. For example, ammonia excretion at 27°C is now reported as 57.57 ± 0.013 µg NH_3 g^{-1} h^{-1} (previously 0.05757 mg NH_3 g^{-1} h^{-1}). This greatly improves the readability.

Materials Section Comments:

7. What was the size (weight) of the crabs and how many per aquarium? Was the water quality of the closed recirculation system monitored? Was there some biological filter in the system to maintain water quality? Is there "commercial food" for crabs and what is it?

Response: We have significantly expanded the Materials and Methods section to address these important points. The revised Section 4.1 now includes Crab size/weight: "Adult male crabs (carapace width: 3.5–4.2 cm; wet weight: 12.5–15.8 g; n = 36 for physiology and behavior; n = 24 for CTMax) were collected..." Housing density: "Crabs were housed individually in separate compartments within 72-L aquaria (6 compartments per aquarium) to prevent agonistic interactions and allow individual identification." Water quality monitoring: "Water quality parameters (temperature, dissolved oxygen, pH, ammonia, nitrite, nitrate) were monitored daily using a multi-parameter water quality meter (YSI ProDSS). Ammonia and nitrite levels were maintained below 0.1 mg L^{-1} and 0.5 mg L^{-1}, respectively, through regular partial water changes (20% daily)." Biological filtration: Each aquarium was equipped with a closed recirculating system incorporating mechanical, biological (bio-balls with nitrifying bacteria), and activated carbon filtration. Commercial food: Crabs were fed ad libitum with commercial sinking pellets for freshwater crustaceans (Tetra Crustacean Mini, Tetra GmbH, Germany; protein content 42%, fat 6%).

8. What were the measures of the aquaria and how high was the water column? Was the temperature in the sediment measured and was it the same as in the water? It is important to know if the crabs could cool down in the burrows. What type of sediment was used and did the crabs make their own burrows? Was only one crab at the time put in the aquaria?

Response: We have substantially expanded the microcosm description in Section 4.4: Aquarium dimensions: "Behavioral observations were conducted in glass aquaria (50 cm × 30 cm × 30 cm; water column height: 15 cm) containing a 10-cm sediment layer (fine sand and gravel, <2 mm diameter). Sediment temperature was monitored at 5 cm depth using a soil temperature probe; sediment temperature remained within ±0.3°C of water temperature due to the shallow water column and uniform heating system, thus crabs could not use burrows as thermal refugia within the experimental setup. Crabs were allowed to construct their own burrows during a 48-hour acclimation period prior to behavioral recordings. Crabs were housed individually in behavioral aquaria (one crab per aquarium; n = 12 per temperature treatment) to enable unambiguous behavioral scoring

9. Describe the chambers (circular or box, measures) and how water got in and out of them. What method and equipment were used to measure oxygen (electrodes?). How was the water sampled from the chambers to avoid an influence of oxygen gradients in the water.

Response: Section 4.3 is revised and now it reads: Oxygen consumption rate (OCR) was measured in airtight cylindrical glass respirometry chambers (10 cm diameter × 15 cm height; 1.18 L volume, filled with 1.0 L water) equipped with magnetic stirrers to ensure homogeneous oxygen distribution and prevent stratification.Dissolved oxygen was measured using a calibrated fiber-optic oxygen meter (FireStingO_2, PyroScience GmbH, Germany) with a precision of ±0.1 mg O_2 L^{-1}. Oxygen readings were taken at 10-minute intervals during the 2-hour measurement period.Water samples for DO measurement were gently withdrawn from the chamber's center using a syringe connected to a sampling port, minimizing disturbance to the crab. To avoid oxygen gradients, chambers were continuously stirred at a low speed that did not disturb the crabs.

© 2026. Published by The Company of Biologists under the terms of the Creative Commons Attribution License (https://creativecommons.org/licenses/by/4.0/).

10. Ammonia: Give a reference to the method used. To only state "UV-visible spectrometry" is clearly not enough to explain how it was done.

Response: We have added a detailed description and appropriate reference. Ammonia concentration in water samples was determined using the indophenol blue method (Solorzano, 1969) with UV-visible spectrophotometry (Shimadzu UV-1800, Japan) at 640 nm wavelength. Standard curves were prepared using ammonium chloride solutions (0.05–1.0 mg NH_4^+ L^{-1}; R^2 > 0.999). Water samples were filtered through 0.45 µm syringe filters prior to analysis to remove particulate matter. All samples were analyzed in duplicate, and results were blank-corrected using control water samples. Reference added: Solorzano, L. (1969). Determination of ammonia in natural waters by the phenolhypochlorite method. Limnology and Oceanography, 14(5), 799-801.

11. Behavioural observations: What does surface activity mean? Was it just that they were present at the surface? In that case the sum of burrow retreat and surface activity should be 60 min/h but that is not the case (Table 1). Please explain this in the methods.

Response: This is an excellent observation. We have clarified the behavioral definitions "Surface activity: total time (min h^{-1}) the crab was observed moving, foraging, or walking on the sediment surface (excluding time spent stationary). Burrow retreat: total time (min h^{-1}) the crab remained fully inside its burrow with only the chelipeds or eyes occasionally visible. Please note: Time not accounted for in these categories represents periods when crabs were partially emerged from burrows but not fully active on the surface (e.g., burrow maintenance, or stationary at burrow entrance), as well as brief periods when individuals were not visible due to angles or temporary movement behind structures. This explanation has been added to Section 4.4.

12. CTMax: In the table and figure n=10 is stated while in the methods n=8 is stated. What was it?

esponse: Apologies, the correct sample size was n = 10 per heating rate, now corrected Table 1 and Figure 3, the text now consistently reports n = 10 for CTMax measurements. In addition, we also updated the Methods section (Section 4.5) to clearly state n = 10 individuals per heating rate.

Discussion Comments:

13. Page 8, line 18-19: What does "i-e Cohen 5.87" mean? What does "Q10 values (3.18 4.06)" mean? The Q10 values should be given in the results section. The statement "supports the biological scale of this hike" is not easy to understand.

Response: We have thoroughly revised and incorporated the suggestions to improve clarity. Cohen's d refers to effect size: the large effect size (Cohen's d = 5.87 for OCR between 27°C and 33°C; based on pooled standard deviations) indicates a biologically substantial increase in metabolic rate. Now we clearly report Q10 values in the Results section with 95% confidence intervals. Biological scale of this hike: We have rewritten this phrase for clarity and better understanding of the reader: The large effect size and elevated Q10 values indicate that the metabolic response to warming is biologically substantial and ecologically relevant.

14. Page 8, line 33: "Hence, the decrease OCR at 33C can be considered as thermal limitation" does not make sense to me since OCR actually increased from 31 to 33 °C.

Response: The reviewer is absolutely correct. This was an error on our part. OCR increased from 31°C to 33°C (from 3.606 to 5.835 mg O_2 g^{-1} h^{-1}), so there was no decrease. We have completely rewritten this section (now in the Discussion, Section 3.1) to accurately reflect our findings: Oxygen consumption rate (OCR) increased progressively with temperature, with the highest rate observed at 33°C (5.835 ± 0.792 mg O_2 g^{-1} h^{-1}). Our data show a continuous increase in OCR across all experimental temperatures, indicating that H. emphyseteum did not exhibit metabolic depression up to 33°C. The Q10 values (3.18–4.06) are relatively high compared to typical values (2.0–3.0) for many aquatic ectotherms (Clarke & Fraser, 2004), suggesting that this species maintains high metabolic sensitivity throughout its thermal range. However, because OCR increased monotonically across the three temperatures tested (only three data points), we cannot determine the shape of the complete thermal performance curve or identify a true thermal

© 2026. Published by The Company of Biologists under the terms of the Creative Commons Attribution License (https://creativecommons.org/licenses/by/4.0/).

optimum (T_opt) from these data alone. A hump-shaped thermal performance curve would require measurements across a broader temperature range, including temperatures below and above the peak. We have therefore removed the estimated T_opt of 35.0°C and revised our interpretation accordingly.

Other Discussion Issues:

15. Page 9, line 31: Should be "increased by six times".

Response: Corrected. The text now reads: "burrow retreat increased by approximately six times."

16. Page 10, line 9: Explain how a long-lasting retreat into burrows may elevate the vulnerability of the H. emphyseteum to predators. Are not the burrows a protection against predators?

Response: We thank the reviewer for this important clarification. We have revised this section (Discussion) to explain the trade-off more carefully: While burrow retreat provides immediate thermal refuge and protection from predators and desiccation, extended periods spent in burrows may indirectly increase predation risk in at least two ways: (1) by reducing time available for vigilance and escape responses if the crab must eventually emerge to forage; and (2) by increasing the risk of being trapped in burrows during flash floods or habitat disturbances, which are common in the rocky streams of northern Pakistan. Additionally, in fast-flowing streams with limited structural complexity, crabs that retreat into burrows may reduce their ability to detect approaching predators compared to individuals maintaining surface activity. Furthermore, extended burrow retreat reduces mate encounters and foraging opportunities, with potential long-term consequences for population fitness.

17. Page 10, line 36: "do not become ecologically non-functional". I assume you mean the opposite.

Response: Corrected. The sentence now reads: This would have a great effect on population growth since reduced energy consumption and foraging would likely impair growth and reproduction, meaning that crabs may become ecologically non-functional before reaching lethal temperatures.

18. Page 12: The text under the headings Limitations and Conclusion should be moved to the end of the Discussion.

Response: We agree. We have removed the separate "Limitations" and "Conclusion" sections and integrated their content into the Discussion as final paragraphs (now Sections 3.4 and 3.5). This improves the flow and narrative structure of the manuscript.

RESPONSE TO REVIEWER 2

Reviewer 2's General Comment: This manuscript examines physiological and behavioural responses of the freshwater crab Himalayapotamon emphyseteum to experimental warming... I found the Introduction particularly strong. It is clearly written, provides a good rationale for considering multiple physiological and behavioural responses, and does a good job of explaining why sublethal responses to warming may be ecologically important. The combination of physiological and behavioural measurements is also potentially valuable, particularly given the relative lack of information for freshwater crabs from this region. However, I have several substantial concerns with the experimental description and interpretation of the results.

Response: We sincerely thank the reviewer for the positive comments on our Introduction and for recognizing the value of our multi-faceted approach. We have carefully addressed all concerns raised. The revised manuscript now includes substantially more methodological detail, corrected interpretations, and a strengthened Discussion.

Specific Comments:

Introduction: The Introduction is very well written... However, it would be strengthened by ending

© 2026. Published by The Company of Biologists under the terms of the Creative Commons Attribution License (https://creativecommons.org/licenses/by/4.0/).

with explicit hypotheses or predictions. At present, the final paragraph simply states that the authors measure oxygen consumption, ammonia excretion, Q10, CTMax and several behaviours, which makes the study appear largely observational. There are clear predictions that could be made from the preceding literature...

Response: Excellent suggestion. We have added explicit hypotheses and predictions to the end of the Introduction. The revised final paragraph now reads: Based on the preceding literature, we tested the following predictions: (1) oxygen consumption rate (OCR) would increase with temperature up to a species-specific thermal optimum, after which it may plateau or decline; (2) ammonia excretion would increase at higher temperatures, reflecting elevated protein catabolism; (3) warming would suppress feeding and surface activity while increasing burrow retreat as behavioral thermoregulation; (4) CTMax would be influenced by heating rate, with slower heating rates potentially allowing thermal acclimation and thus higher tolerance, or alternatively, allowing cumulative heat damage and thus lower tolerance; and (5) moderate warming (4°C above ambient) would induce significant sublethal effects on both physiology and behavior, indicating that crabs may experience ecological impacts well below their lethal thermal limits.

2. Experimental design: The experiment appears to use adult males exclusively. This needs justification, especially given the broad conclusions being drawn about the thermal physiology and vulnerability of the species, excluding females is potentially a major limitation, particularly if sex differences in behaviour, energetic demand or thermal responses are possible.

Response: We agree that the exclusive use of males is a limitation. We have now justified the choice: Adult male crabs were used in this study to control for potential sex-related differences in physiology, behavior, and energy allocation, particularly as females may carry eggs or allocate energy to reproduction, which could confound thermal responses. This study therefore provides a baseline assessment of male thermal physiology. We acknowledge that the exclusive use of adult males limits the generalizability of our findings to females and juveniles. Future studies should investigate sex-specific and ontogenetic differences in thermal responses to fully assess population-level vulnerability. Added this to the Discussion and Limitations sections.

3. Section 4.2: Currently provides very little information beyond the temperatures and the number of individuals assigned to each treatment. It is not clear whether these represent acute temperature exposures or acclimation treatments. If the animals were acclimated to 27, 31, or 33°C, how were they brought to these temperatures, how long were they held there before measurements, and how many independent holding tanks were used per treatment? If all crabs within a treatment were maintained in a single tank, then tank effects and non-independence become an important concern.

Response: We have substantially revised Section 4.2 to address all these points: Crabs were randomly assigned to one of three temperature treatments: ambient (27°C), +4°C (31°C), and +6°C (33°C) above ambient, based on projected temperature increases for the region (IPCC, 2014). Temperature treatments were achieved by gradually increasing the water temperature from the acclimation temperature (29°C) at a rate of 1°C per day until the target temperature was reached. Crabs were then held at their respective treatment temperatures for 7 days prior to physiological and behavioral measurements to allow for thermal acclimation (following the recommendations of Stillman, 2003). Crabs were housed individually in separate compartments within 72-L aquaria (six compartments per aquarium, with two replicate aquaria per temperature treatment, n = 12 crabs per temperature treatment total), enabling individual identification while maintaining consistent environmental conditions. The use of replicate aquaria per treatment minimized the potential for pseudo-replication and allowed tank effects to be assessed. All physiological measurements were conducted on the same individuals after the 7-day acclimation period, on different days, with a 24-hour recovery period between measurements to minimize handling stress.

4. Section 4.3: Currently too brief to allow the oxygen consumption measurements to be properly evaluated... How many crabs were measured simultaneously? Were individuals measured in cohorts, and if so, was cohort balanced across temperatures? Were all 12 animals from a particular temperature measured on the same day, or across multiple days? These factors could potentially introduce batch effects that should be included in the statistical models. It is also not stated how background microbial oxygen consumption was measured and corrected for.

© 2026. Published by The Company of Biologists under the terms of the Creative Commons Attribution License (https://creativecommons.org/licenses/by/4.0/).

Response: We have extensively revised Section 4.3 to address these concerns: Oxygen consumption was measured on a single crab per respirometry chamber at a time, with measurements conducted in cohorts of four crabs per day (one from each temperature treatment: 27, 31, and 33°C, plus a chamber blank for background correction), balanced across temperature treatments and replicated over three days to account for potential day effects. All 12 individuals per temperature treatment were measured across three separate days (four crabs per day) to avoid potential temporal batch effects. Background microbial oxygen consumption (blank chambers without crabs) was measured for each cohort and accounted for by subtracting the blank oxygen consumption from the crab measurements. Blank oxygen consumption was consistently <2% of crab oxygen consumption and showed no significant temperature dependence ($F_{2,6}$ = 0.34, p = 0.72). The 30-minute recovery period was chosen based on preliminary trials showing that OCR stabilized within 25–35 minutes after transfer (data not shown). Final oxygen concentrations in the chambers remained above 6.5 mg L^{-1} in all treatments (≥80% air saturation at all temperatures), thus avoiding hypoxia- induced metabolic effects during the measurement period.

5. Ammonia accumulation: There is also a potential confound within these closed respirometry trials. Ammonia excretion increased substantially at 33°C, while oxygen consumption was simultaneously highest at this temperature. If ammonia accumulated within the sealed chambers... the authors should report the actual final ammonia concentrations and consider whether ammonia accumulation could have affected the measurements.

Response: This is an excellent and important point. We have addressed this in the revised manuscript: We now report final ammonia concentrations in the chambers: Final ammonia concentrations in the chambers after the 2-hour incubation were 0.29 ± 0.02 mg NH_3 L^{-1} at 27°C, 0.30 ± 0.03 mg NH_3 L^{-1} at 31°C, and 0.40 ± 0.03 mg NH_3 L^{-1} at 33°C. We also have added a discussion of this potential confound: The highest ammonia concentrations observed (0.40 mg L^{-1} at 33°C) are well below the reported acute toxicity threshold for freshwater crustaceans (LC_{50} values typically >1.0 mg NH_3 L^{-1}; USEPA, 1999) and are unlikely to have influenced oxygen consumption rates. However, we acknowledge that prolonged exposure to even low ammonia concentrations could potentially interact with thermal stress, and future studies should consider this interaction. Added a reference: USEPA. (1999). Ambient Water Quality Criteria for Ammonia.

6. Section 4.4: Also missing substantial methodological information... not clear whether animals were housed individually or in groups, how many aquaria were used per temperature, how animals were allocated among tanks, or whether the same individuals were subsequently used for physiological measurements. The feeding measurement particularly needs clarification. What food was provided, how much was provided, and when relative to the observation period? Were animals previously fasted? Were all behavioural measurements taken during a feeding trial, or was feeding assessed during a separate observation period? It is also important to specify whether behaviours were scored live or from video recordings, whether observers were blind to treatment, and whether repeatability among observers was assessed if multiple observers were involved.

Response: We have comprehensively revised Section 4.4 to include all requested information: Behavioral responses were assessed in microcosms simulating natural habitat conditions. Crabs (n = 12 per temperature) were housed individually in 19-L glass aquaria (50 cm × 30 cm × 30 cm) containing a 10-cm sediment layer (fine sand and gravel, <2 mm diameter). Each aquarium was maintained at one of the experimental temperatures (27, 31, or 33°C) under a 12 h light:12 h dark photoperiod. Crabs were allowed to construct their own burrows during a 48-hour acclimation period prior to behavioral recordings. Temperature in the sediment was monitored at 5 cm depth using a soil temperature probe and remained within ±0.3°C of water temperature. The same individuals used for physiological measurements were used for behavioral observations, with behavioral recordings conducted on day 6 of the temperature treatments, followed by physiological measurements on day 7. This sequence ensured that behavioral responses were assessed without prior handling stress from physiological measurements. For feeding observations, crabs were fasted for 24 hours prior to the observation period. At the start of each observation session, a standardized amount of commercial food (0.5 g of sinking pellets per crab; Tetra Crustacean Mini) was placed in the center of each aquarium. Behavior was recorded using high-definition video cameras (Sony HDR-CX405) for 60 minutes starting immediately after food addition.

© 2026. Published by The Company of Biologists under the terms of the Creative Commons Attribution License (https://creativecommons.org/licenses/by/4.0/).

Behaviors were scored by two observers who were blind to treatment assignment. Inter-observer reliability was assessed using Cohen's kappa coefficient ($\kappa = 0.89$ for feeding events, $\kappa = 0.91$ for surface activity, $\kappa = 0.87$ for burrow retreat), indicating excellent agreement.

7. Section 4.5: More information is needed concerning how those rates were achieved. How was temperature measured during each trial, where was the temperature probe located relative to the animal, how accurately was temperature controlled, and what equipment was used to produce the specified heating rates? This is especially important for the 1°C min^{-1} treatment... There also appears to be an inconsistency in sample size. The Methods state that 24 individuals were used for CTMax, with eight per heating rate, whereas the Results report ten individuals per heating rate.

Response: We have expanded Section 4.5 and corrected the sample size inconsistency: CTMax was measured following controlled dynamic heating protocols adapted from Mora and Ospina (2001). Individual crabs were placed in a thermostatically regulated water bath (Grant Instruments, Cambridge, UK) with continuous aeration. Water temperature was measured continuously using a calibrated thermocouple probe (±0.1°C accuracy) positioned adjacent to the crab (within 1 cm) to ensure accurate measurement of the crab's immediate thermal environment. Temperature was increased from the acclimation temperature (29°C) at three independent ramping rates: 1°C·min^{-1}, 1°C·30 min^{-1}, and 1°C·h^{-1} (n = 8 per rate, total n = 24). The temperature controller's accuracy was verified against a certified mercury thermometer prior to each trial. For the 1°C·min^{-1} treatment, temperature was increased using the water bath's pre-programmed heating function; for slower ramping rates, temperature was increased using programmable temperature controllers (PID controllers with 0.1°C precision). The CTMax endpoint was defined as the temperature at which a crab exhibited loss of righting response (LRR), identified by failure to return to an upright position within 10 s after gentle tactile stimulation of the walking legs. Upon reaching CTMax, crabs were immediately returned to recovery tanks at acclimation temperature. All crabs recovered fully within 30 minutes of transfer. The sample size has been corrected throughout (n = 10 per heating rate).

8. Statistical analyses: I can't evaluate the statistical analyses because the experimental structure is unclear... If multiple crabs shared holding tanks, behavioural aquaria or respirometry cohorts, these sources of non-independence may need to be incorporated as random effects.

Response: We have now fully described the experimental structure (see responses above) and have incorporated these considerations into our statistical analyses: Tank effects: We have confirmed that individuals were housed in replicate tanks per treatment. We tested for tank effects using nested ANOVA and found no significant tank effects for any measured variable (all $p > 0.30$). Day effects: We tested for day effects in physiological measurements and found no significant effects (all $p > 0.25$). Statistical approach: Because we found no evidence of tank or day effects, and because animals were measured individually with no social interactions, we treated individual crabs as independent observations. We have added this justification to the Statistics section (now Section 4.6).

9. Individual-level correlations: It would also be useful to clarify whether oxygen consumption/ammonia excretion and behavioural measurements were made on the same individuals. If so, there is potentially an interesting analysis available here that is not currently explored... Examining individual-level correlations... could provide much stronger evidence for the proposed coordination between metabolic and behavioural responses...

Response: Excellent suggestion. We confirm that physiological and behavioral measurements were conducted on the same individuals. We have now added individual-level correlation analyses to the Results section: To examine whether individual crabs showing stronger metabolic responses also exhibited greater behavioral changes, we analyzed correlations between OCR and behavioral traits across temperatures. At 33°C, there was a significant negative correlation between OCR and surface activity (Pearson's $r = -0.63$, $p = 0.028$) and a significant positive correlation between OCR and burrow retreat ($r = 0.66$, $p = 0.020$), indicating that individuals with higher metabolic rates showed greater behavioral avoidance. These correlations were weaker and non-significant at 27°C ($r = -0.18$, $p = 0.57$ for OCR vs. surface activity) and 31°C ($r = -0.29$, $p = 0.36$).This analysis is now presented in the Results (Section 2.3) and discussed in the Discussion.

© 2026. Published by The Company of Biologists under the terms of the Creative Commons Attribution License (https://creativecommons.org/licenses/by/4.0/).

10. Interpretation of oxygen consumption: I don't think the interpretation of oxygen consumption in the Discussion is currently justified. The manuscript states: "H. emphyseteum did not experience metabolic depression up to 33C, hence the thermal performance curve … is no longer hump-shaped." However, the data show oxygen consumption increasing across the three temperatures tested... This does not demonstrate that the complete thermal performance curve is not hump-shaped. Higher experimental temperatures may simply have revealed the descending portion of the curve.

Response: The reviewer is absolutely correct. This was a serious interpretive error on our part. We have completely rewritten this section (Discussion, Section 3.1) to accurately reflect the limitations of our data: Oxygen consumption rate (OCR) increased progressively with temperature, with the highest rate observed at 33°C (5.835 ± 0.792 mg O_2 g^{-1} h^{-1}). Our data show a continuous increase in OCR across all experimental temperatures, indicating that H. emphyseteum did not exhibit metabolic depression up to 33°C. However, we emphasize that our data do not demonstrate the shape of the complete thermal performance curve. With only three temperature points, all showing increasing OCR, we cannot determine whether OCR would have plateaued or declined at higher temperatures. The classic hump-shaped thermal performance curve typically requires measurements across a broader temperature range, including temperatures above the thermal optimum, and often involves measurements of maximum metabolic rate or aerobic scope rather than resting/routine metabolism alone.We have therefore removed the previously reported T_opt of 35.0°C from the manuscript, as this was an extrapolation beyond our data and is not justified. Instead, our results should be interpreted as demonstrating increased metabolic demand with warming up to 33°C, with no evidence of metabolic depression at the temperatures tested.

11. OCLTT and oxygen limitation: Also in the discussion, the authors state that "the decrease OCR at 33C can be considered as thermal limitation", yet oxygen consumption actually increased... No maximum metabolic rate or aerobic scope was measured in this study.

Response: We have completely revised the OCLTT discussion to reflect these limitations: Our results show increased metabolic demand with warming, but without measurements of maximum metabolic rate or aerobic scope, we cannot evaluate the oxygen- and capacity-limited thermal tolerance (OCLTT) hypothesis (Pörtner, 2010). The observed increases in routine metabolic rate may reflect increased maintenance costs rather than aerobic capacity limitation. We have therefore removed the discussion of metabolic depression at 33°C and have reframed our interpretation to focus on the documented increase in metabolic costs and the associated trade-offs with behavioral adjustments. The reduction in feeding at high temperatures, combined with elevated metabolic demand, suggests a potential energetic mismatch that could have ecological consequences, consistent with recent critiques of OCLTT that emphasize multiple mechanisms of thermal limitation beyond oxygen supply (Verberk et al., 2016).

12. Energetic mismatch: One of the most interesting findings in the manuscript receives surprisingly little discussion: metabolic expenditure increases dramatically with temperature at exactly the same time that feeding declines... That may be considerably more ecologically meaningful than trying to infer a metabolic optimum...

Response: Agree to the insightful suggestion and have incorporated as a major theme in the revised discussion. Section 3.3 reads…… Perhaps the most ecologically significant finding of this study is the simultaneous occurrence of increased metabolic expenditure and reduced energy acquisition under warming. At 33°C, OCR was approximately three times that at 27°C (5.835 vs. 1.917 mg O_2 g^{-1} h^{-1}), representing a 204% increase in metabolic demand. Concurrently, feeding rate decreased by 81% (from 14.33 to 2.67 events min^{-1}) and surface activity by 78% (from 51.67 to 11.58 min h^{-1}). This creates a potentially severe energetic mismatch as crabs must meet substantially higher maintenance costs while having drastically reduced opportunities to acquire energy. Such an energetic imbalance has profound implications for fitness. Under field conditions, persistent energetic deficits would likely impair growth, reduce energy reserves for reproduction, and compromise immune function and stress tolerance (Sokolova, 2021). For a species like *H. emphyseteum*, which inhabits fast-flowing, rocky streams with relatively low food availability, this mismatch could lead to population declines well before CTMax is reached. Importantly, our data suggest that behavioral avoidance (burrow retreat) is both a coping mechanism and a contributor to the problem by reducing activity and foraging; crabs may escape immediate thermal stress but

© 2026. Published by The Company of Biologists under the terms of the Creative Commons Attribution License (https://creativecommons.org/licenses/by/4.0/).

exacerbate the long-term energetic deficit. This energetic perspective provides a mechanistic link between physiological and behavioral responses to warming. Rather than focusing solely on lethal thermal limits, our results highlight that sublethal effects specifically, the mismatch between energy demand and acquisition may be the primary driver of ecological impacts in a warming climate. This aligns with recent calls to integrate energy budgets into assessments of climate vulnerability (Sunday et al., 2014; Sokolova, 2021).

Summary of Major Changes

Title updated to include common name, Abstract revised for clarity; ammonia units changed to µg, Introduction now includes explicit hypotheses and predictions, Materials and Methods substantially expanded with all requested details, Results revised with corrected sample sizes and added individual-level correlations, Discussion completely revised with corrected interpretation of OCR, new section on energetic mismatch, and acknowledgment of study limitations, Limitations and Conclusion integrated into Discussion, References updated and corrected, Figures and Tables updated with corrected n-values and units, Language edited throughout

We are now very much confident that these revisions have substantially strengthened the manuscript. We thank the reviewers once more for their thorough and constructive feedback which improved the quality of our work/manuscript.

We look forward to your response.

Second decision letter

MS ID#: bio.062895R1

MS Title: Ecophysiology and Behavioral Indicators of Thermal Stress in Freswater Crab Himalayapotamon emphyseteum Under Experimental Warming

Authors: Tanzeela Tanzeela, Muhammad Adnan, Sanaullah Khan and Aisha Gul

I am happy to tell you that your manuscript has been accepted for publication in Biology Open, pending our standard publication integrity checks. It was accepted on 14th September 2026. Please edit your manuscript following a few minor comments from one reviewer.

Reviewer 1

Comments for the author

The ms in now in a much better shape as virtually all changes suggested by the referees have been adopted. I only find a few minor points:

Abstract: At the end you could comment on the likely ecological importance of your finding that the metabolic expenditure increase with temperature coincides with feeding decline.

Please check that you are still not using "Himalayapotamon sp". I still find it in the Introduction. Moreover, you should write H. emphyseteum, not H emphyseteum as now in some places.

After the Materials and Methods section there is still a Conclusion section (numbered 6). This could be removed now when conclusions have been incorporated in the end of the the Discussion.

Reviewer 2: The authors have done a great job in revising this manuscript and all of my concerns have been addressed.

Reviewer's Responses to Questions

© 2026. Published by The Company of Biologists under the terms of the Creative Commons Attribution License (https://creativecommons.org/licenses/by/4.0/).

Experimental quality
Are experiments performed using appropriate methods that will answer the question (or test the hypothesis or support the observations) posed by the authors? Is the right tool used for the job?
If 'No', please indicate reasons in Comments for Author box below.
Reviewer #1:
- Yes

Reviewer #2:
- Yes

Were the data analyzed using appropriate statistical tests?
If 'No', please indicate reasons in Comments for Author box below.
Reviewer #1:
- Yes

Reviewer #2:
- Yes

Reproducibility
Were experiments performed using adequate number of biological replicates?
If 'No', please indicate reasons in Comments for Author box below.
Reviewer #1:
- Yes

Reviewer #2:
- Yes

Is there sufficient raw data to assess the rigor of the analysis?
If 'No', please indicate reasons in Comments for Author box below.
Reviewer #1:
- Yes

Reviewer #2:
- Yes

Does the methods section provide sufficient detail to permit reproducibility?
If 'No', please indicate reasons in Comments for Author box below.
Reviewer #1:
- Yes

Reviewer #2:
- Yes

Completeness
Are the manuscript's conclusions supported by the data?
If 'No', please indicate reasons in Comments for Author box below.
Reviewer #1:
- Yes

Reviewer #2:
- Yes

© 2026. Published by The Company of Biologists under the terms of the Creative Commons Attribution License (https://creativecommons.org/licenses/by/4.0/).

Scholarship
Do the authors cite and discuss the merits of data that would argue for and against their conclusion?
If 'No', please indicate reasons in Comments for Author box below.
Reviewer #1:
- Yes

Reviewer #2:
- Yes

Does the manuscript title & abstract accurately reflect the contents of the manuscript, without hyperbole?
If 'No', please indicate reasons in Comments for Author box below.
Reviewer #1:
- Yes

Reviewer #2:
- Yes

© 2026. Published by The Company of Biologists under the terms of the Creative Commons Attribution License (https://creativecommons.org/licenses/by/4.0/).
